# Lithic Techno-economics and OSL Chronology of the Later Stone Age at Chessungalane, Save River Valley, Mozambique

**DOI:** 10.1007/s10437-026-09659-7

**Published:** 2026-06-06

**Authors:** Osama Samawi, Jeffrey I. Rose, Li Li, Ana Gomes, Daniel Martins, Elena Skosey-LaLonde, Gina M. Buckley, Ilona Benedetti, J. Michael Daniels, Jan-Pieter Buylaert, Jonathan A. Haws, Juma Nacuti Turquia, May Murungi, Michael M. Benedetti, Milena Carvalho, Milton Chirindza, Mussa Raja, Profina Júlio Mondlane, Sarah Marie Foley, Takalani A. Dubayi, Telma João Sipaneque, Nuno Bicho

**Affiliations:** 1 ICArEHB – The Interdisciplinary Centre for Archaeology and Evolution of Human Behaviour, Universidade do Algarve, Campus de Gambelas, 8005-139 Faro, Portugal; 2 Key Laboratory of Vertebrate Evolution and Human Origins, Institute of Vertebrate Paleontology and Paleoanthropology, Chinese Academy of Sciences, Beijing, 100044 China; 3 Department of Human Origins, Max Planck Institute for Evolutionary Anthropology, Deutscher Pl. 6, 04103 Leipzig, Germany; 4 Centre for Marine and Environmental Research (CIMA)—Infrastructure Network in Aquatic Research (ARNET), Faculty of Science and Technology, University of Algarve, Faro, Portugal; 5 University of North Carolina Wilmington, Wilmington, NC USA; 6 University of Denver, Denver, CO USA; 7 Baylor University, Waco, TX USA; 8 University of Louisville, Louisville, KY USA; 9 Universidade Eduardo Mondlane, Maputo, Mozambique; 10 Human Evolution Research Institute, University of Cape Town, Rondebosch, 7700 South Africa; 11 Department of Earth and Ocean Sciences, University of North Carolina Wilmington, Wilmington, NC USA; 12 Department of Physics, Technical University of Denmark, 4000 Roskilde, Denmark

**Keywords:** Late Stone Age, Lithic refits, Raw material economy, Lithic technology, Mozambique

## Abstract

Nestled between East and Southern Africa, the Stone Age archaeology of Mozambique offers important insights into Later Stone Age (LSA) behavioral variability across a region pivotal to understanding human adaptations. Here, we present results from technological analysis, refitting and optically stimulated luminescence (OSL) dating of the newly discovered site of Chessungalane (CHE) in south-central Mozambique. OSL dates indicate occupation spanning ~ 23–7 ka, corresponding to the early LSA through Wilton periods. Technological analysis of lithic artifacts, refitting, and raw material survey reveals differential technological investment strategies employed by LSA groups. Chert was introduced during last occupation phase for systematic bladelet production through complex reduction sequences, likely due to its scarcity and superior knapping qualities. In contrast, quartz and quartzite were procured during all the occupation phases for simple flake production via shorter, simpler reduction sequences, reflecting their local abundance and fracture constraints. Preliminary comparison with contemporaneous LSA sequences from the broader region suggests that CHE followed a distinct technological trajectory, with bladelet production appearing significantly later in time, highlighting the importance of regional variability in shaping LSA technological behavior. The results demonstrate a strong relationship between raw material availability, quality, and technological investment, and contribute critical new data to the largely undocumented LSA record of Mozambique.

**Supplementary Information:**

The online version contains supplementary material available at https://doi.org/10.1007/s10437-026-09659-7.

## Résumé

Situé entre l'Afrique orientale et l'Afrique australe, le Mozambique offre, grâce à ses vestiges archéologiques, des informations importantes sur la variabilité comportementale à la fin de l'âge de pierre (LSA) dans une région essentielle à la compréhension des adaptations humaines. Nous présentons ici les résultats de l'analyse technologique, des remontages et de la datation par luminescence stimulée optiquement (OSL) du site récemment découvert de Chessungalane, dans le centre-sud du Mozambique. Les dates OSL indiquent une occupation s'étendant il y a environ 23 000 à 7 000 ans, ce qui correspond à la période allant du début du LSA à la période Wilton. L'analyse technologique des artefacts lithiques, les remontages et l'étude des matières premières révèlent les différentes stratégies d'investissement technologique employées par les groupes du LSA. Le chert a été introduit au cours de la dernière phase d'occupation pour la production systématique de lamelles à travers des séquences de réduction complexes, probablement en raison de sa rareté et de ses qualités supérieures pour la taille. En revanche, le quartz et le quartzite ont été utilisés pendant toutes les phases d'occupation pour la production simple d'éclats à travers des séquences de réduction plus courtes et plus simples, reflétant leur abondance locale et leurs contraintes de fracture. Une comparaison préliminaire avec les séquences contemorais du LSA (Late Stone Age) de la région élargie suggère que Chessungalane a suivi une trajectoire technologique distincte, la production de lamelles apparaissant nettement plus tardivement, ce qui souligne l'importance de la variabilité régionale dans le façonnement des comportements technologiques du LSA. Les résultats démontrent une relation étroite entre la disponibilité des matières premières, leur qualité et l'investissement technologique, tout en apportant de nouvelles données critiques à la documentation encore largement lacunaire du LSA au Mozambique.

## Introduction

Raw material selection represents the first physical manifestation of the mental template underlying lithic production (Bar-Yosef & Van Peer, 2009). The availability and fracture characteristics of lithic raw materials have fundamentally shaped both the spatial distribution of past human settlements (Brantingham, 2003, 2006) and the technological strategies available to hunter-gatherers (Will, 2021). Understanding raw material selection within a techno-economic framework provides crucial insights into hunter-gatherer mobility patterns, technological decision-making, and adaptive resilience (Adams & Blades, 2009; Andrefsky, 1994; Kuhn, 1994; Nelson, 1991; Will et al., 2026). Here we examine these techno-economic practices during the pivotal transition from the Pleistocene to the Holocene in Southern Mozambique.

The LSA in Africa marks a fundamental shift in human technological organization and social complexity, characterized by the widespread adoption of microlithic tools, diversification of raw material procurement strategies, and increasingly elaborate exchange networks (Ambrose, 1998; Mitchell, 1988). Chronologically, the LSA spans Marine Isotope Stages (MIS) 3 to 1, approximately 40,000 to 2000 years ago (Lombard et al., 2012), a period during which hunter-gatherer populations navigated dramatic environmental fluctuations and developed new economic practices, including the eventual adoption of herding in some regions.

**Fig. 1:**
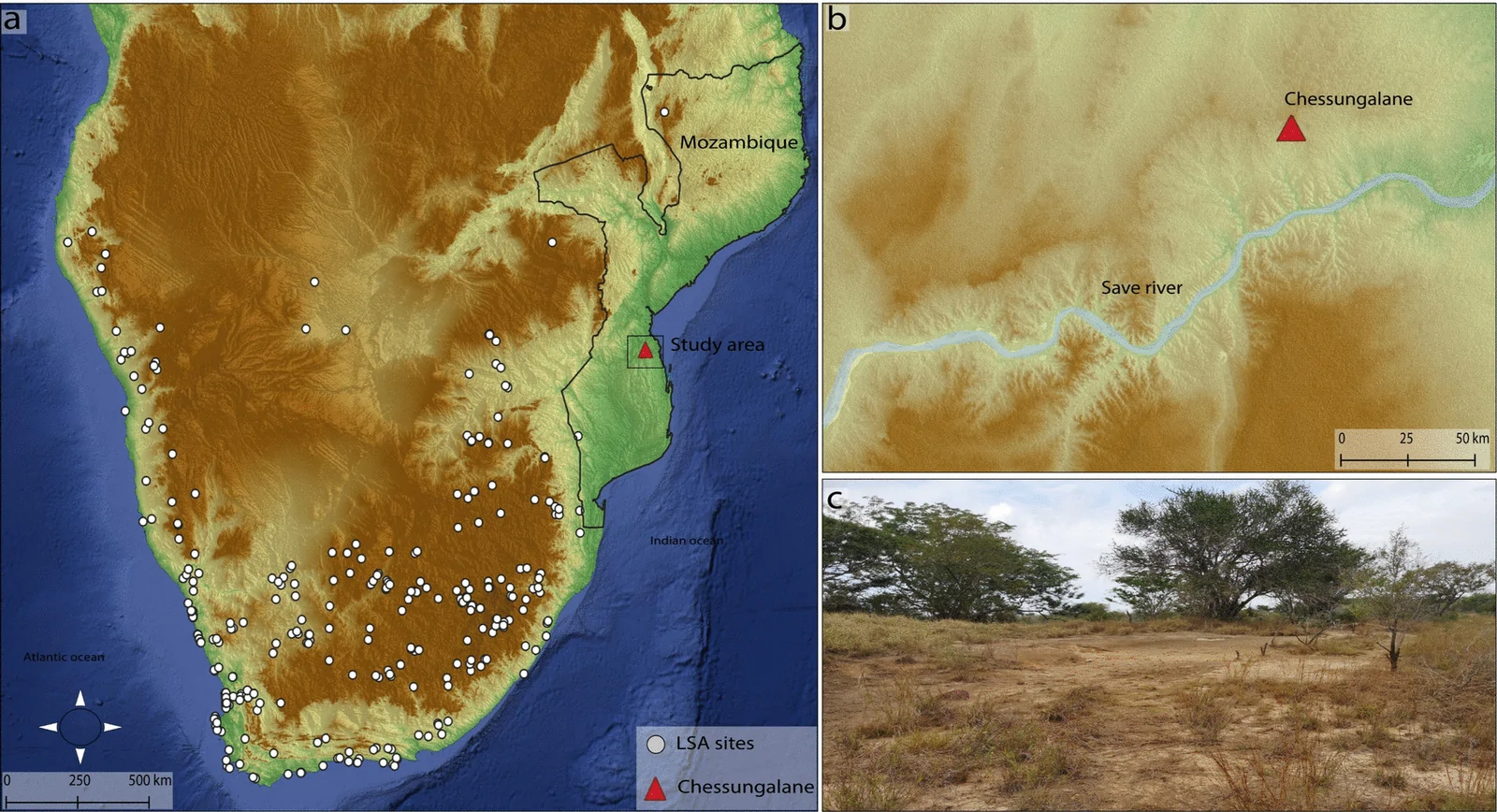
Location and environmental context of Chessungalane. **a** Map of Southern Africa showing the distribution of LSA sites (Hallinan, 2026; Loftus et al., 2019) and the location of Chessungalane in south-central Mozambique. **b** Regional context showing CHE’s location relative to the Save River in Inhambane Province, south-central Mozambique. **c** Site landscape showing the tropical savanna environment with scattered acacia trees and grassland vegetation typical of the coastal plain

Southern Africa preserves the most extensive and well-studied LSA archaeological record on the continent (Fig. 1a). Here, the LSA sequence is traditionally divided into six techno complexes (Bicho, 2021; Hallinan, 2026), defined primarily through lithic techno-typology and raw material selection patterns. The early major techno complexes are the Early LSA and the Robberg, marking the clear end of the preceding Middle Stone Age (MSA) behaviour. The Early LSA (ca. 40–20 ka) is characterized by a noticeable increase in the adoption of bipolar techniques for micro-flake production on predominantly local raw materials, with a rare bladelet component (Bader et al., 2022). The nature and geographic extent of the ELSA remain debated, with recent work questioning whether it represents a single widespread tradition originating from Border Cave in South Africa or regionally variable responses to local conditions (Bousman & Brink, 2018). The subsequent Robberg technocomplex (ca. 20–12 ka) is defined by the innovation of systematic bladelet production on fine-grained raw materials using both freehand and bipolar techniques, with generally low frequencies of retouched tools (Porraz et al., 2016), and recent studies suggest that the ELSA-Robberg transition was gradual, with bladelet technology refining incrementally pushed by natural or human parameters (Blessing et al., 2023).

Later LSA phases reveal dynamic technological trajectories. The Oakhurst technocomplex (ca. 12–7 ka) shifts toward macrolithic, flake-based production using simple cores with minimal preparation, preferentially selecting hornfels and quartzite presetting a break in past laminar production such as Bushman Rock Shelter show (Thomas, 2022). This gives way to the Wilton technocomplex (ca. 8–4 ka), marked by standardized microlithic production, including backed tools, on diverse raw materials, as seen at Rose Cottage Cave (Guillemard, 2024). The final LSA (ca. 4–0.1 ka) and ceramic LSA (< 2 ka) witness declining formalization of lithic technology, with production relying increasingly on simple cores, informal tools, and greater emphasis on locally available materials (Sampson, 1974).

Moving beyond nomenclature and typology, the behavioral variability documented across these six technocomplexes reflects complex interactions among environmental change, resource availability, demographic shifts, and the transmission of technological knowledge. The Southern African LSA sequence, however, has been predominantly defined by sites in South Africa, leaving substantial geographic gaps in our understanding of regional variability. Mozambique, positioned at the intersection of tropical savanna and coastal environments, represents a critical, yet understudied, region for examining LSA technological diversity. The present study addresses this gap through analysis of the Chessungalane (CHE) (Fig. 1b) site in south-central Mozambique discovered by the DISPERSALS project, providing the first well-dated LSA sequence in the coastal Save region.

Primary observations from the CHE assemblages reveal different patterns of raw material consumption and technological practices across at least three occupation phases. As the first LSA site in Mozambique with detailed technological analysis and OSL chronology, CHE provides a critical opportunity to examine past human behavior in a region where the LSA remains poorly documented. This study addresses two main research questions:

1. Chronology and occupation history: What is the chronological framework for LSA occupation at CHE, and how do the assemblages relate to broader LSA technocomplexes?
2. Raw material economy and technological organization: How did raw material availability and quality influence technological investment and reduction strategies at CHE?

## Study Area

The Republic of Mozambique is located in the southeastern part of the African continent and comprises a land surface of about 800,000 km^2^ (Fig. 1a). Geographically, it occupies a strategic position between two key regions of the continent for human evolution: Eastern Africa and Southern Africa (Bicho et al., 2016; Clark, 1966). Topographically, in the north, the provinces of Niassa and Tete have elevations exceeding 2000 m above mean sea level and receive comparatively higher precipitation. By contrast, the central and southern provinces (Sofala, Inhambane, and Gaza) lie at lower altitudes, with rainfall varying across the region but generally increasing toward the southeastern coast.

The present landscape of Mozambique is diverse with coastal plains, savannah, woodlands, and mountains. Several major rivers flow from the west to the Indian Ocean in the east, including the Zambezi, the Limpopo, and the Save. Paleoclimatic studies during the LSA in central Mozambique show cool and wet climatic conditions during the Last Glacial Period, with a shift from forest to grassland ecosystems between c. 22.5 and 7.2 cal kBP, followed by increase in temperature c. 7.2 cal kBP (McWethy et al., 2016).

Archaeological research in Mozambique began during the twentieth century, resulting in the documentation of numerous sites (Santos Júnior, 1950). Investigations dedicated to the Stone Age only gained sustained momentum in the early twenty-first century, through both local and international projects (for an overview, see Gonçalves et al., 2016). Systematic research started in the early 2000 s focusing mainly on the north of the country in the Niassa region, setting the current basis of knowledge for the country’s Stone Age record, shedding light on past human adaptation in this underexplored part of Africa (Mercader, 2009; Mercader et al., 2009, 2012). Starting in 2010, our team expanded the geographic scope of Stone Age research in Mozambique, conducting fieldwork across the north, center, and south of the country and discovering new Stone Age landscapes (Bicho et al., 2016, 2026). Alongside these international initiatives, local researchers have contributed to advancing the understanding of past human adaptations strategies, with particular focus on technological behavior and raw material procurement strategies (Jeiamba, 2021; Muianga, 2025; Munguambe, 2024; Tembe, 2018).

While several general lithic studies have been published, they primarily focus on Middle Stone Age contexts (Bicho et al., 2018a, 2018b; Mercader et al., 2008), with only a limited number of stratified LSA sites examined. The Mozambican LSA broadly follows continental technological trends of the period. Lithic miniaturization, alongside the introduction of ceramics and symbolic ornaments, is present (Bicho et al., 2016). However, to date, no detailed technological analysis has been conducted on any LSA assemblages in Mozambique. This constitutes a significant regional gap, excluding Mozambique from comparative studies and broader syntheses in African Stone Age archaeology.

### Chessungalane

CHE is an open-air site located in south-central Mozambique (− 21°02′18″S 34°31′01″E), named after the nearby village (Fig. 1b). It was discovered in 2023 and excavated in the 2024 field season. The site is situated atop a flat-topped hill approximately 12 km north of the Save River (Fig. 1b), directly overlying a Miocene gravel deposit (Real, 1966), that likely served as a primary raw material source (Fig. 3).

**Fig. 2:**
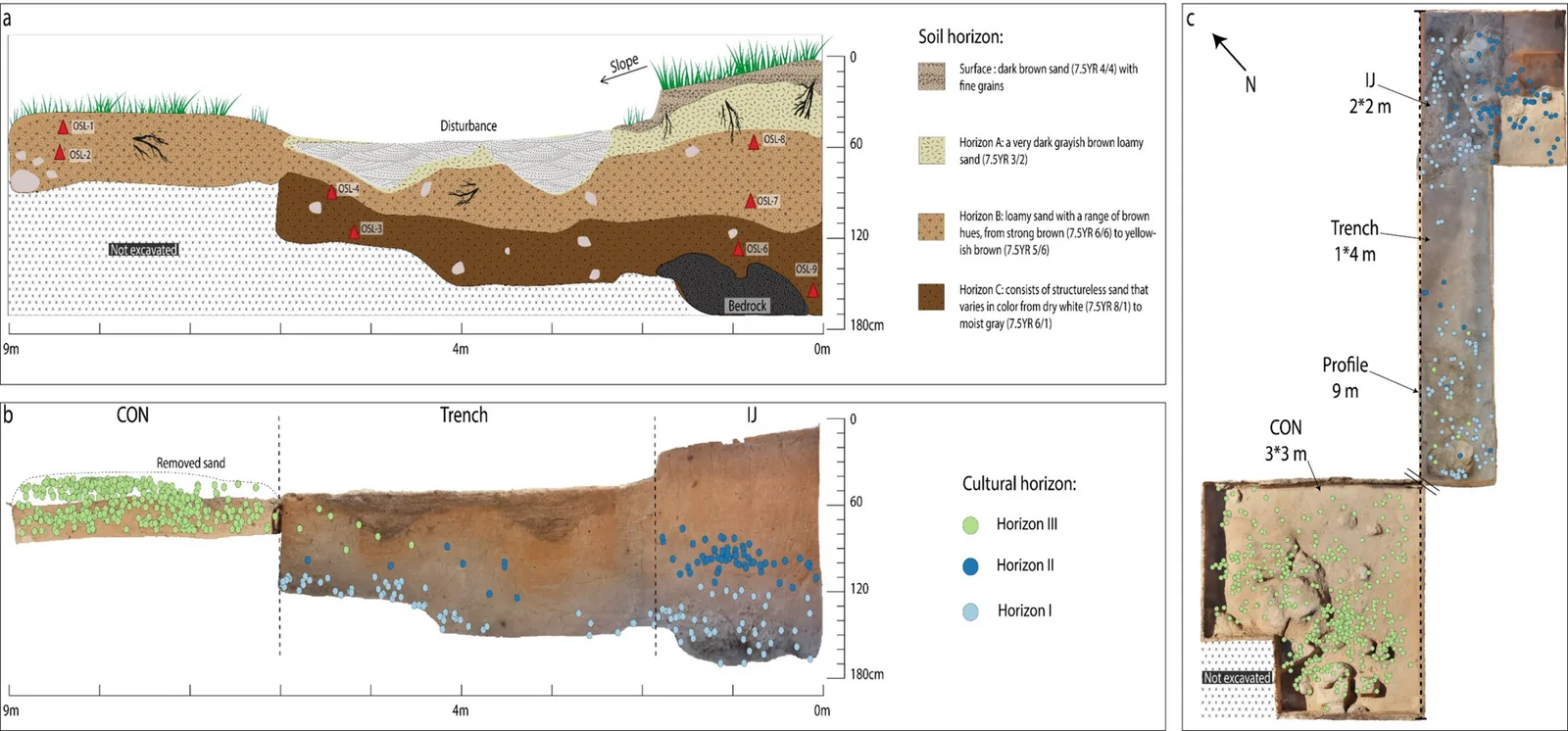
Stratigraphic profile and cultural horizons at Chessungalane. **a** Composite stratigraphic profile showing soil-horizons across the excavated areas. **b** Cultural horizon distribution showing artifact distribution. **c** Excavation area and plan-view photographs

Three primary excavation areas, CON, Trench, and IJ, were opened at CHE, encompassing a total area of 16 m^2^ (Fig. 2). The IJ area was the only one to reach bedrock at a maximum depth of 172 cm (Fig. 2b and c). Evidence of modern anthropogenic disturbance was limited to the uppermost layer of the site (Fig. 2a). No faunal material or ceramics were recovered from the excavated volume of CHE.

### Geoarchaeological Context

Four soil horizons were identified in excavation area IJ following USDA soil taxonomy (Fig. 2a). Soil color was determined in the field using the Munsell Soil Color Charts (Munsell soil Color chart, 2018). The C horizon consists of structureless sand that varies in color from dry white to moist gray, representing minimally altered parent material that directly overlies the local Miocene sandstone and conglomerate bedrock. The B horizon is composed of loamy sand with a range of brown hues, from strong brown to yellowish brown, representing subsoil development with only minimal evidence of root activity. Above lies the A horizon, a very dark grayish brown loamy sand representing an organic-rich topsoil with evidence of recent anthropogenic alteration. The uppermost deposit consists of dark brown sand with fine grain, interpreted as colluvial material derived from recent landscape preparation, likely for local agriculture.

### Archaeological Horizons

While the geoarchaeological sequence distinguishes three primary soil stratigraphic units (A–C), analysis of artifact distribution and technology reveals more complex cultural patterning. The C unit contains a single archaeological horizon (Horizon I), while the B unit encompasses two discrete cultural horizons (Horizons II and III). Horizon I (H-I) represents the earliest occupation, accounting for 33.9% of the total assemblage (*n* = 722 artifacts). Horizon II (H-II), located in the lower portion of the B unit, constitutes a technologically distinct and low-density phase at 9.75% (*n* = 208 artifacts). Horizon III (H-III), occurring in the upper section of the B unit, marks the final and most intensive occupation, comprising 56.4% of the assemblage (*n* = 1203 artifacts). These cultural horizons provide a framework for interpreting technological change and occupation intensity (Fig. 2b).

## Methods and Materials

Excavations at CHE were divided into three excavation areas, employing two total stations for 3D artefact provenance. The basic vertical units followed geological layers whenever they were visible. Within each layer, 5-cm artificial spits were used to assist excavators, and all artefacts above 5 mm were piece-plotted in three dimensions. Exceptions were made for smaller complete bladelets and retouched tools, which were always plotted regardless of size. In addition to 3D plotting, horizontal control was maintained using the bucket approach (McPherron & Dibble, 2002; McPherron et al., 2005), in which a 10-L bucket serves as the excavation unit to control excavated volume and minimize issues of spatial distribution. All excavated sediments were dry-sieved through 3-mm mesh to maximize artefact recovery. Individual labels printed with barcodes were assigned to each artefact to simplify subsequent analyses and curation (www.oldstoneage.com). A total of 2133 lithic artifacts were recovered and subjected to techno-typological analysis following standardized attribute criteria (SI, Table S3) along with refitting studies and raw material surveys.

### OSL Dating

Eight OSL samples were collected from CHE across units CON, Trench, and IJ for chronological control of the LSA occupation sequence (Fig. 2a). Samples were collected by hammering steel tubes into a freshly cleaned profile. Two samples were taken from unit CON (soil-stratigraphic unit B, Horizon III), two from the Trench unit (soil-stratigraphic units B-C, Horizons II—III), and four from unit IJ (soil-stratigraphic units A-C, Horizons II—III). Samples were processed at the Nordic Laboratory for Luminescence Dating at the Technical University of Denmark (DTU). Both multi-grain quartz SAR OSL and feldspar SAR post-IR IRSL techniques were used to date the samples (Buylaert et al., 2012; Murray et al., 2021). Details on the luminescence dating procedures are given in Supplementary Information.

### Raw Material Surveys

**Fig. 3:**
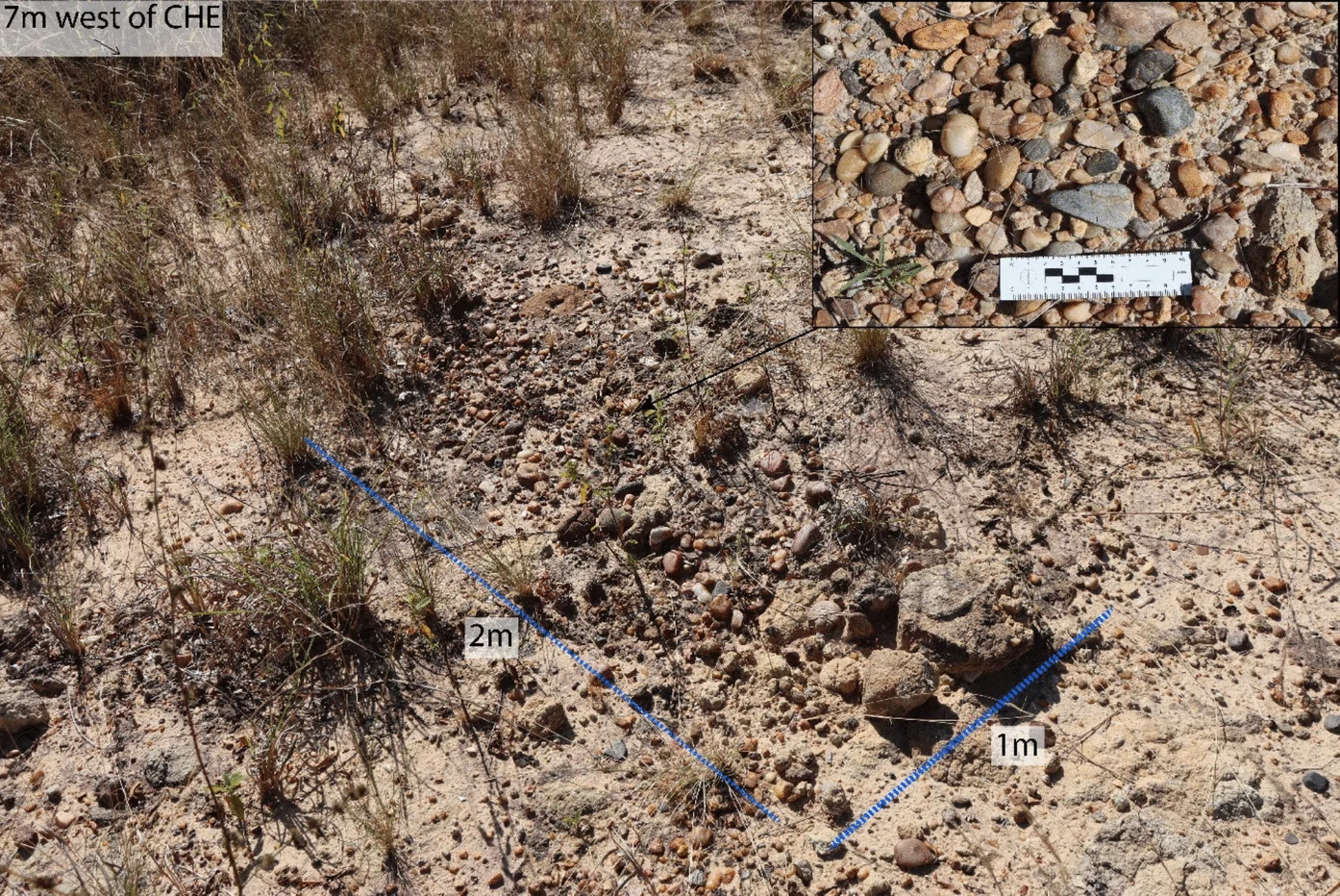
Raw material concentrations next to Chessungalane

CHE is situated atop a consolidated Miocene gravel deposit with pebbles that form the bedrock to the archaeological sequence. The Mozambique Basin in central and Southern Mozambique contains extensive Miocene sedimentary formations, including the Inharrime, Temane, and Jofane Formations, which comprise terrigenous deltaic deposits from the Limpopo and Zambezi rivers (Salman & Abdula, 1995; Lächelt, 2004). These Miocene formations include gravel-bearing horizons that are weathered and eroded to produce the surface pebble concentrations observed at CHE. The site features a pebble outcrop that currently provides only minor surface concentrations of knappable raw material (Fig. 3). A systematic collection was conducted in two 2 × 1 m units to record raw material types and dimensions. Pebbles larger than 20 mm were collected, yielding 40 specimens from the 4 m^2^ survey area.

### Technological and Typological Analysis

To establish a robust analytical framework, attribute analysis was used to quantitatively integrate both typological and technological traits. This provided a structured dataset for examining lithic variability. Complementing this, the *chaîne opératoire* or lithic reduction sequence approach (Holmes, 1894; Leroi-Gourhan, 1964), was used to interpret these traits beyond their metric dimensions, allowing for a reconstruction of reduction sequences and insight into the techno-economic choices made by past human groups (Bar-Yosef & Van Peer, 2009). Together, these methods offer a comprehensive understanding of the production strategies, bridging statistical patterning with behavioral interpretation.

The dominance of quartz in our study area necessitated adjustments to the standard flint-based analytical system. Techno- and typological classification in lithic studies were mostly developed on fine raw material “Flint thinking” (Will, 2021, and Knutsson, 2014), which remains an ongoing challenge for regions with less fracture-predictable materials. A combination of lithic attributes is particularly necessary when working with materials that do not strictly follow conchoidal fracture mechanics (de Lombera Hermida, 2009), such as quartz, as different criteria are required to confidently recognize as anthropogenic (Spry et al., 2022). In the present study, radial cracks, and impact points, were recorded as present or absent exclusively for quartz artifacts. Despite these additional criteria, a degree of analytical error is expected when working with quartz assemblages (Driscoll, 2011; Proffitt & de la Torre, 2014).

Knapping technique is among the most important technological attributes used to characterize LSA techno complexes, and criteria to identify bipolar and freehand percussion were systematically recorded in this study. Bipolar reduction involves placing a core on an anvil and striking it with a hammerstone, producing blanks through the compression of force between the hammer and the anvil (Shea, 2013). A variety of attributes were established (Pargeter et al., 2019), but in this study, complete blanks produced by this technique are identified only by opposed impact points, crushed platforms, irregular termination, and extensive battering on core surfaces. Freehand percussion involves holding a core in the hand and striking it with a hammerstone. Products of this technique do not exhibit bipolar attributes but instead feature a bulb of percussion, intact platforms, and occasionally lipping and diffuse bulbs when a soft hammer was used such as sandstone, bone, or wood.

The lithic assemblage was classified into five main categories: debitage, tools, cores, shatter, and unworked material. Debitage encompasses all detached artifacts from a core (e.g., flakes, blades, bladelets) produced during the reduction sequence. Special attention was given to core-trimming elements (CTEs), which are debitage products that reflect technological strategies for rejuvenating and maintaining cores (Barham, 1989; Shea, 2013).

Following the *chaîne opératoire* framework, three reduction stages were identified within the CHE assemblages. The initialization stage includes cortical debitage with cortical platforms, representing the initial removals following raw material selection. The shaping stage includes crested blades/bladelets and naturally backed knives (NBK), used to establish narrow flaking surfaces for bladelet production. The maintenance stage includes core tablets (striking platform removals), rejuvenation flakes, and overpassed flakes, all produced to correct knapping errors and maintain reduction efficiency.

Cores are volumes of raw material exhibiting at least one extraction. They were categorized based on their intended production goals: blade or flake-proportionate blanks. Laminar cores were designed to produce elongated blanks from either prepared or cortical platforms, often through semi-rotating or fully rotating extraction strategies. In contrast, flake cores were oriented toward the production of oval-shaped blanks, detached from prepared or cortical platforms across one or multiple working surfaces. To assess the reduction intensity among different reduction systems and raw materials in CHE, Scar Density Index (SDI) was calculated following Clarkson (2013), by dividing the number of flake scars greater than 10 mm by the estimated surface area of each core, calculated as 2 × (length × width + width × thickness + length × thickness).

Shatter in this study encompasses non-diagnostic debris smaller than 10 mm, as well as angular fragments larger than 10 mm. In archaeological contexts dominated by quartz-based industries, high frequencies of shatter are expected. This is primarily due to the brittle nature of quartz, which often results in unpredictable fractures during knapping (Spry et al., 2022) or blank modification. Unworked objects at CHE were either unstruck pebbles or hammerstones showing a clear battered area. Tools were identified by intentional regular modification using only general typologies: scrapers, notches, truncations, backed pieces, burins, or simple retouch pieces.

### Lithic Refitting and Spatial Analyses

Lithic artifacts provide insights into past technological and economic behaviors, but only a snapshot at the final stage of reduction or time of discard (Dibble et al., 2017). Refitting studies are a powerful tool for reconstructing multiple stages of the reduction sequence, reaching back to early lithic specialists of the late 1800 s (Spurrell, 1880). Despite their analytical potential, there are a lack of refit studies in African LSA research (Chiotti et al., 2007; Phillips, 1988; Pruvost et al., 2025), and to the best of our knowledge, are largely absent from LSA studies in Southern Africa, with only seven refits reported from the late Holocene site of Buterix 1, Eastern Cape (Guillemard, 2025). This gap likely reflects the considerable amount of time that the method requires, and researchers’ interests.

At CHE, refitting followed the *chaîne opératoire* framework, guided by technological and raw material attributes. Lithic artifacts were first organized by raw material type and colors. Artifacts larger than 1 cm from each horizon were then grouped by raw material type, followed by an analysis of their technological features. Following the methodology of Cziesla (1990), we established two primary refit categories to interpret the assemblage: production sequence refits (PSR) and break refits (BR).

- Production sequence refits (PSR) directly reflect the intentional designs and reduction strategies of the knappers. Sub-categories were created for a finer analytical resolution, including:
  o Flake-to-flake (FF): Conjoining the ventral surface of one flake to the dorsal surface of another.
  o Flake-to-core (FC): Linking a flake’s ventral surface to its originating core.
- Break refits (BR) provide equally informative insights, but they primarily speak to post-depositional processes and site integrity rather than reduction strategies. These included:
  o Split flakes (SF): Joining multiple fragments of a single blank.
  o Split pebbles (SP): Conjoining unmodified pebble fragments.

The spatial distribution of refitted artifacts has been a major focus in lithic studies for decades (Cahen & Moeyersons, 1977). Such analyses are critical for reconstructing site use through horizontal patterning (e.g., activity areas) and for evaluating post-depositional processes and site integrity through vertical patterning (Möller et al., 2025; Sisk & Shea, 2008). We examined the spatial distribution of refitted artifacts at CHE, with a particular emphasis on vertical movement. We included only refitting sequences containing more than three artifacts in the spatial analysis. To assess clustering across horizons, we calculated Moran’s I spatial autocorrelation. Moran’s I measure spatial autocorrelation, indicating whether nearby values form clusters (positive), dispersion (negative), or randomness (0) (Waldhör, 1996). For detailed representation of reduction sequences, all refits associated with cores were digitally documented using 3D photogrammetry, processed in Metashape, and visualized in Blender.

## Results

### OSL Dates

Eight quartz OSL samples from CHE yielded ages spanning approximately ~ 23 to ~ 5 ka, covering late Marine Isotope Stage (MIS) 2 through the mid-Holocene (Table 1). The stratigraphically consistent dates establish a chronological framework for the LSA occupation sequence, though one sample (OSL 4 from unit trench) shows a stratigraphic inversion and is excluded from chronological interpretation. While stratigraphic consistency supports the reliability of the overall sequence, bleaching confidence varies among samples (see Supplementary Tables S1 and S2), and ages other than OSL 3 were used cautiously.

**Table 1:** OSL dating results from Chessungalane

Depth (cm)	Lab code	Sample no.	Area	Soil Horizon	Cultural Horizon	OSL age ka	Status^*^
10	252322	OSL-1	CON	B	Horizon III	6.7 ± 0.4	Accepted
31	252323	OSL-2	CON	B	Horizon III	8.6 ± 0.5	Accepted
53	252325	OSL-4	Trench	B	Horizon II	15.5 ± 0.9	Rejected
61	252328	OSL-8	IJ	B	Horizon II	4.7 ± 0.3	Accepted
87	252324	OSL-3	Trench	B/C	Horizons I and II	10.6 ± 0.6	Accepted
109	252327	OSL-7	IJ	B	Horizon II	7.8 ± 0.4	Accepted
150	252326	OSL-6	IJ	B/C	Horizons I and II	11.5 ± 0.6	Accepted
184	252329	OSL-9	IJ	C	Horizon I	23.3 ± 1.7	Accepted

*The “Status” column indicates whether each OSL age was accepted or rejected archaeologically based on stratigraphic consistency (age-depth ordering within the depositional sequence), rather than individual sample quality indicators such as bleaching assessment or error size (See Supplementary Information for more detail)

The deepest sample from unit IJ, OSL 9 (184 cm depth), yielded the oldest age of 23.3 ± 1.7 ka (late MIS 2). Higher in the same unit, OSL 6 (150 cm depth) dated to 11.5 ± 0.6 ka, marking the terminal Pleistocene. OSL 3 from the Trench unit (87-cm depth) gives an age of 10.6 ± 0.6 ka and is thus coeval to sample OSL6. The mid-Holocene occupation is documented in unit CON by OSL 2(31-cm depth, 8.6 ± 0.5 ka) and OSL 1 (10-cm depth, 6.7 ± 0.4 ka). In unit IJ, this occupation is represented by OSL 7 (109-cm depth, 7.8 ± 0.4 ka). OSL8 from unit IJ (4.7 ± 0.3 ka) likely represents the end of Layer A deposition.

Based on general chronological ranges reported from other LSA sites in Southern Africa (Lombard et al., 2012; Mitchell, 2002), the CHE sequence spans from the Early LSA through the Wilton period. Horizon I (~ 23.3 to ~ 10.6 ka) corresponds to the Early LSA in its basal levels, transitioning to Oakhurst characteristics in its upper levels. Horizon II (~ 11.5 to ~ 8.6 ka) falls within the Oakhurst period. Horizon III (~ 8.6 to ~ 6.7 ka) represents the Oakhurst-Wilton transition, characterized by the introduction of systematic bladelet production and backed tools.

### Raw Material Survey

Raw materials are present in low concentrations within the gravel surface deposits surrounding the site (Table 2). Milky quartz dominates, occurring in medium to high quality, followed by grainy quartzite and medium to fine chert. All materials were in pebbles, with average dimensions of 37.7 mm (length), 25.8 mm (width), and 17.7 mm (thickness). Based on their characteristics and local availability, such gravel deposits likely served as primary raw material sources during the occupation phases at CHE.

**Table 2:** Raw material survey results from surface collections from both units

Raw material	No	Avg length (mm)	Avg width (mm)	Avg thickness (mm)	Geomean^*^
Chert	3	41.4	31.1	16.4	27.5
Quartz	27	34.6	24.4	16.6	23.9
Quartzite	10	44.9	28.2	21.2	29.6

*Geometric mean defines shape as the ratio of each dimension to the geometric mean of all dimensions (L × W × T)1/3 (Jungers et al., 1995)

### Lithic Assemblages

The CHE assemblage is dominated by quartz, with lesser amounts of chert and quartzite (Table 3). Raw material proportions remain consistent across H-I and H-II, with a notable increase in chert in H-III. The assemblage is heavily fragmented, with shatter comprising 64.8% and debitage 30.1%, while cores (2.5%), tools (1.3%), unworked material (1.1%), and hard hammers (0.05%) are rare.

**Table 3:** Lithic assemblage composition by horizon and raw material

Category	Horizon I				Horizon II				Horizon III				Total
	Chert	Quartz	Quartzite	Other	Chert	Quartz	Quartzite	Other	Chert	Quartz	Quartzite	Other	
Blade	1	1	-	-	-	3	-	-	39	4	4	-	52
Bladelet	-	-	-	-	-	1	-	-	65	11	7	-	84
Flake	6	92	16	-	3	40	10	-	43	182	22	-	414
Small-flake	-	11	-	-	-	4	-	-	12	20	-	-	47
Splinter	-	5	-	-	-	1	-	-	-	6	-	-	12
**Total debitage**	**7**	**109**	**16**	**-**	**3**	**49**	**10**	**-**	**160**	**223**	**33**	**-**	**610**
Core tablet	-	-	-	-	-	-	-	-	3	-	-	-	3
Crested	-	-	-	-	-	-	-	-	5	-	-	-	5
NBK	-	-	-	-	1	-	-	-	18	-	-	-	19
Rejuvenation	-	-	-	-	-	-	-	-	6	-	1	-	7
**Total CTE**	**-**	**-**	**-**	**-**	**1**	**-**	**-**	**-**	**32**	**-**	**1**	**-**	**34**
Blade	-	-	-	-	1	1	-	-	-	1	1	-	4
Bladelet	1	1	-	-	-	-	-	-	8	1	-	-	11
Flake	-	8	2	-	2	9	-	-	2	13	4	-	40
**Total Core**	**1**	**9**	**2**	**-**	**3**	**10**	**-**	**-**	**10**	**15**	**5**	**-**	**55**
Backed	-	-	-	-	-	-	-	-	7	1	-	-	8
Burin	-	-	-	-	-	-	-	-	-	1	-	-	1
Notch	-	4	1	-	-	2	-	-	-	-	1	-	8
Retouched	-	-	-	-	-	-	-	-	-	1	1	-	2
Scraper	-	1	-	-	-	-	-	-	-	4	-	-	5
Truncation	-	-	-	-	-	-	-	-	-	3	-	-	3
**Total tools**	**-**	**5**	**1**	**-**	**-**	**2**	**-**	**-**	**7**	**10**	**2**	**-**	**27**
Hammerstone	-	-	-	-	-	-	-	-	-	-	1	-	1
Unworked	-	5	1	-	1	3	-	-	-	12	2	-	24
**Total unworked**	**-**	**5**	**1**	**-**	**1**	**3**	**-**	**-**	**-**	**12**	**3**	**-**	**25**
Waste	9	495	56	6	-	104	19	3	37	570	64	20	1383
**Total waste**	**9**	**495**	**56**	**6**	**-**	**104**	**19**	**3**	**37**	**570**	**64**	**20**	**1383**
**Grand total**	**17**	**623**	**76**	**6**	**8**	**168**	**29**	**3**	**245**	**830**	**108**	**20**	**2133**

### Debitage Production

**Fig. 4:**
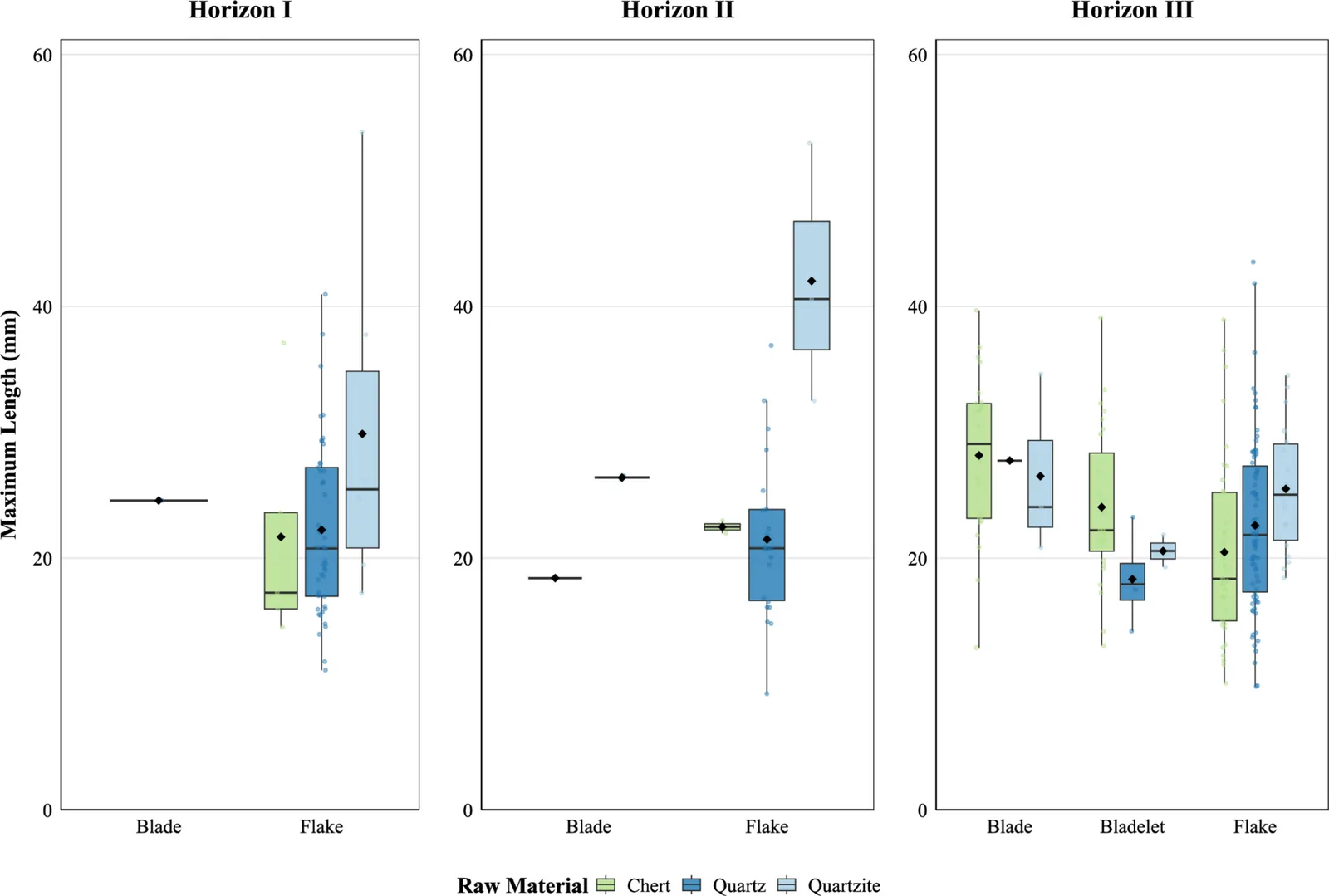
Box plot for the complete debitage maximum length by horizons and raw material

**Fig. 5:**
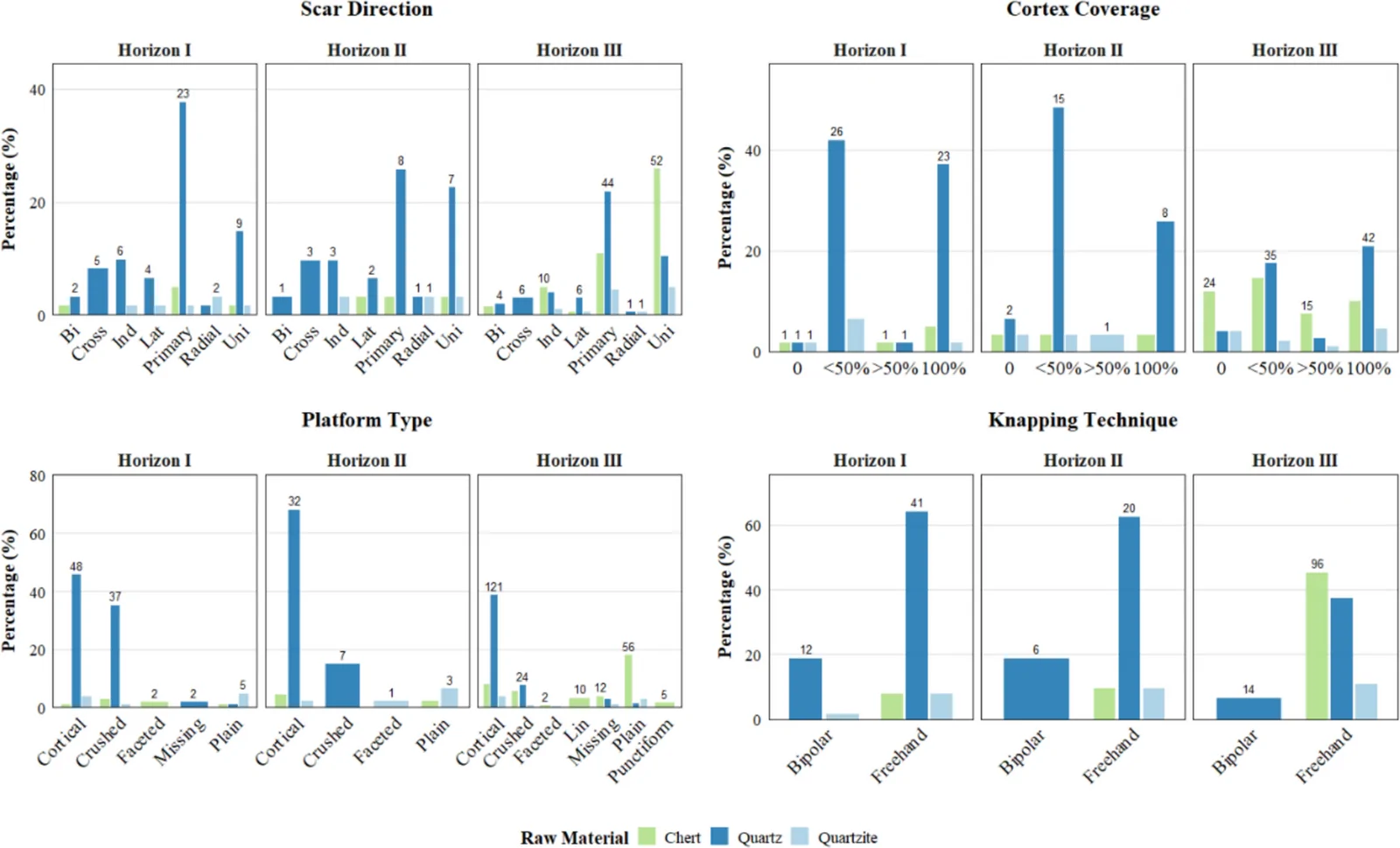
Selected technological attributes of complete blanks by horizon and raw material

Debitage made up 30.1% of the total CHE assemblage, with a higher accumulation in the upper horizons. Across all horizons, quartz flakes were the most common debitage type (Table 3), representing 61.5% of the production. In H-I, quartz flakes comprise 84.4% of the debitage. They were produced primarily using freehand knapping (see Supplementary Fig S1), identified by the absence of opposed impact points and crushed platforms that characterize bipolar reduction. Flakes were relatively small (Fig. 4), with average dimensions of 23 mm (length), 18.5 mm (width), and 7 mm (thickness). A range of exploitation strategies were used to produce flakes in H-I, with a noticeable tendency toward unidirectional flaking from unprepared cortical platforms. The majority of these flakes exhibit semi-cortical coverage to full coverage of cortex with cortical platforms (Fig. 5).

In H II, quartz flakes still dominated, accounting for 82% of the debitage. They were slightly larger on average than those from H-I, with dimensions of 24 mm, 20.1 mm, and 8 mm. The knapping technique remained predominantly freehand, with unprepared platforms and similar scar pattern characteristics with higher percentage of semi cortical coverage (Fig. 5).

A significant shift in production occurred in H-III, with the introduction of Micro-laminar products. Chert bladelets account for 22% of the horizon debitage measuring on average 23.2 mm, 8.2 mm, and 3 mm. Those bladelets were exclusively produced using freehand techniques indicated by 75% of diffused bulbs of percussions with 37.5% of lipped platforms (Table 4). Eighty percent of the chert bladelets were produced using a unidirectional exploitation strategy from prepared platform (Fig. 5).

**Table 4:** Platform lipping and bulb of percussion from complete and proximal pieces across horizons and raw materials

	Horizon I		Horizon II		Horizon III		
	Chert	Quartzite	Chert	Quartzite	Chert	Quartzite	Total
Platform lipping	**7**	**10**	**3**	**5**	**130**	**29**	**184**
Present	1	1	-	-	34	1	37
Absent	4	8	3	5	72	24	116
N/A	2	1	-	-	24	4	31
Bulb of percussion	**7**	**10**	**3**	**5**	**130**	**29**	**184**
Diffused	4	6	-	2	102	23	137
Pronounced	-	2	2	3	3	1	11
Fragmented	-	1	1	-	1	1	4
N/A	3	1	-	-	24	4	32

From the flake debitage in H-III, quartz are counted for 57% of the flakes averaging 22.6 mm, 19.2 mm, and 7.8 mm (Fig. 4). Most flakes were produced using freehand knapping (90%), with 10% showing bipolar-on-anvil features. About 37% retain cortical platforms, and only 20% show crushed platforms due to bipolar production. Quartz flakes are mostly present with semi cortical coverage (less than 50%) with ovate shape with steep cortical lateral edges or fully primary flakes (Fig. 5).

**Fig. 6:**
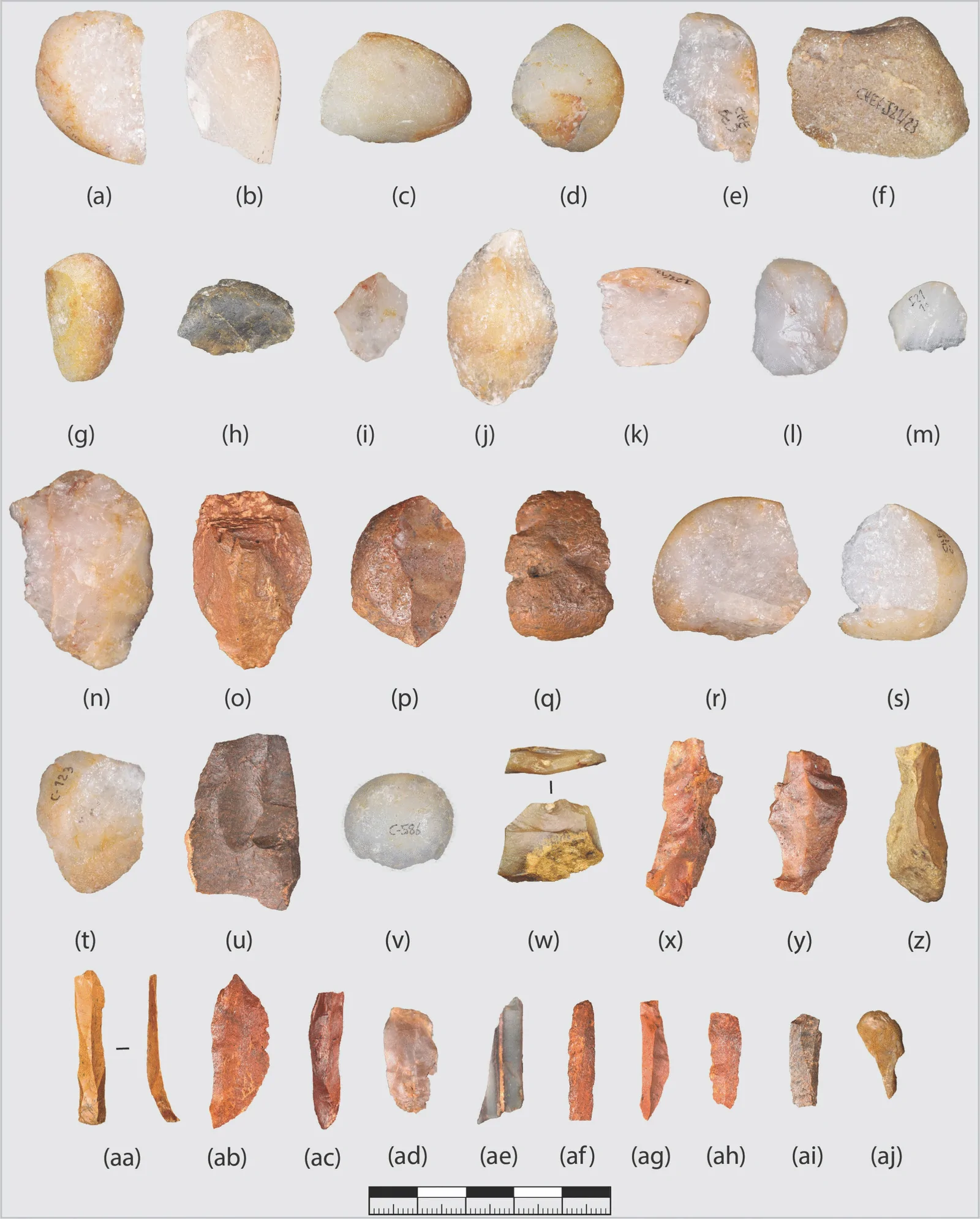
Selected debitage from Chessungalane. **a**–**e** Horizon I; **f**–**m** Horizon II; **n**–**aj** Horizon III. **ab**, **ad**, **af**–**ai** Bladelets; **c**, **d**, **f**–**h**, **q**, **v**, **aj** Cortical flakes (opening flakes); **ac** Crested bladelet; **w** Core tablet; **a**, **b**, **e**, **i**–**n** Flakes; **p**, **x**, **z**, **ae** Naturally backed knives; **aa** Overshot bladelet; **o**, **u**, **y** Rejuvenation flakes. Note: artifacts are oriented platform-up

CTEs represent a minor but technologically significant component of the assemblage, occurring primarily in H-III (Table 3). Forty-three CTEs were identified, concentrated almost entirely on chert (*n* = 32). The majority of CTEs relate to core shaping and consisted of NBKs, also known as ridge flakes (Fig. 6p, x, z, a-e). These 19 pieces exhibit unidirectional dorsal scar patterns. Additionally, five crested bladelets with lateral scars were recorded (Fig. 6ac). Flaking surface maintenance is represented by six core-cleaning or rejuvenation flakes (Fig. 6o, u, y), identifiable by hinge or step terminations on their dorsal surfaces, and a single rejuvenation flake on quartzite. Platform maintenance products are limited to three core tablets (Fig. 6w).

### Core Technology

**Fig. 7:**
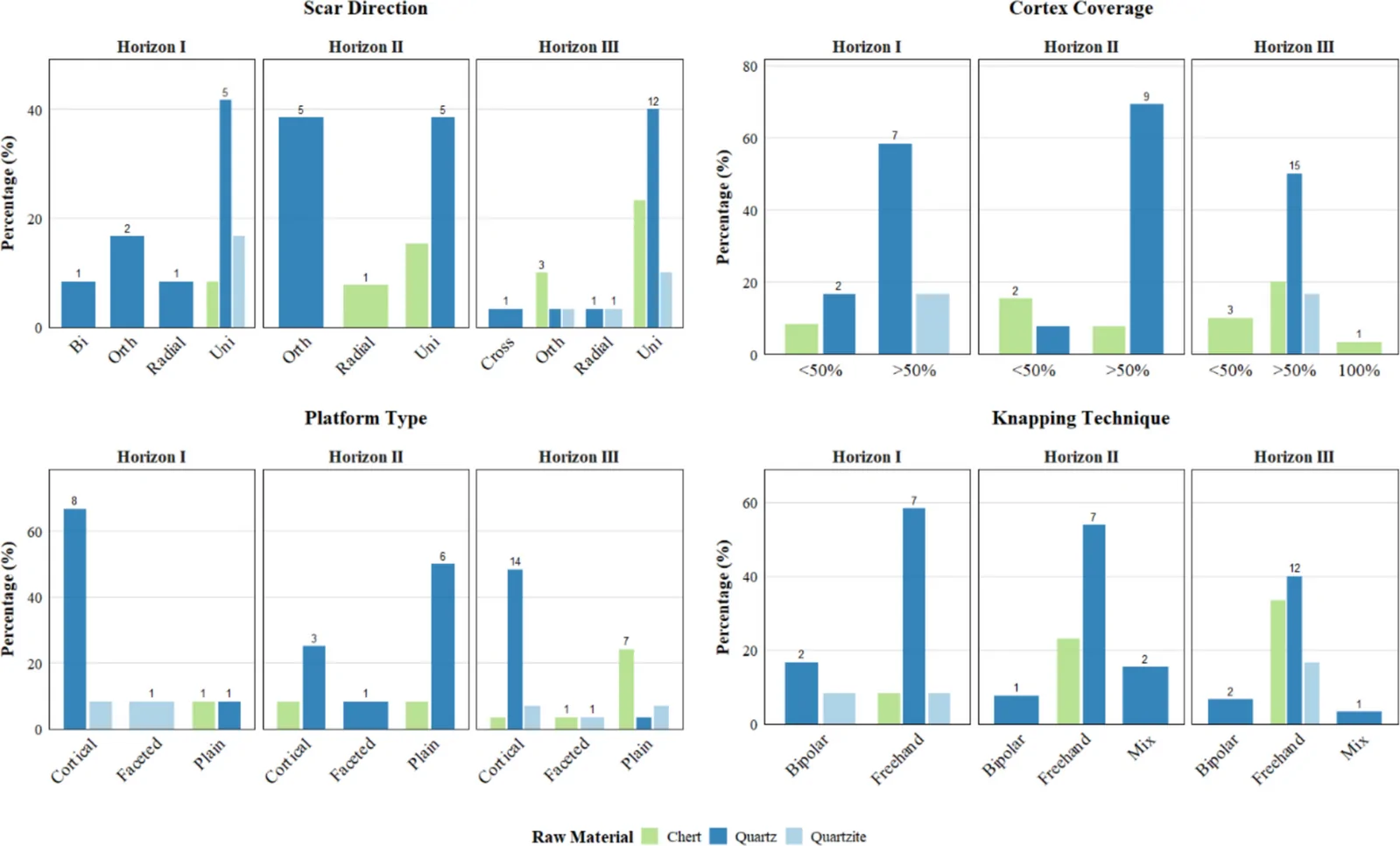
Selected technological attributes of complete cores by horizon and raw material

Cores account for 2.6% of the total CHE assemblage. Flake cores were the dominant type, making up 72% from tall cores (Table 3). The cores are generally small and have similar metric dimensions across all raw materials, although quartzite cores are slightly larger (Table 5). In H-I, Flake Cores were reduced using freehand knapping focused on a single flaking surface initiated from a cortical platform (Fig. 7f and h).

**Table 5:** Average dimensions of the cores by horizons and raw material

Horizon	Raw material	no	Avg length (mm)	Avg width (mm)	Avg thickness (mm)	Last removal length (mm)	Avg of removals	Geometric mean (mm)	Weight (g)
Horizon I	Quartz	9	28.4	24.6	20.2	19.5	2.4	23.7	19.6
	Quartzite	2	34.0	30.1	19.4	31.5	1.0	26.7	32.4
Horizon II	Chert	2	40.6	32.2	24.0	27.0	4.5	31.0	44.1
	Quartz	10	34.4	33.3	25.3	22.0	2.5	29.9	41.0
Horizon III	Chert	10	35.3	29.9	22.0	27.0	3.4	28.0	32.5
	Quartz	15	31.7	29.5	19.4	19.0	1.5	25.9	28.1
	Quartzite	4	57.5	46.4	25.5	24.0	2.0	40.2	124.4

**Fig. 8:**
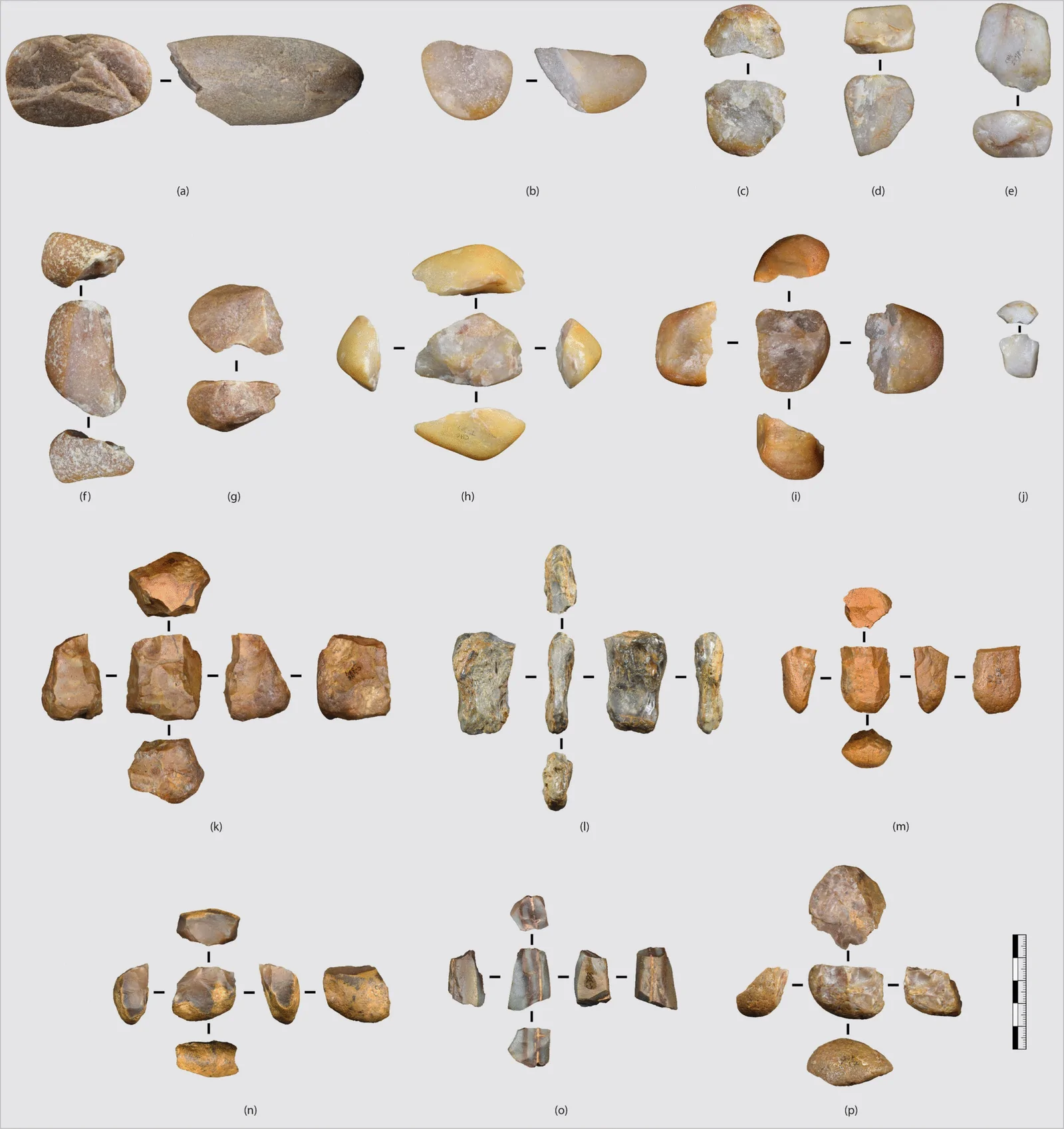
Selected cores from Chessungalane. **f**, **h** Horizon I; **b**, **c**, **e** Horizon II; **a**, **d**, **g**, **i**–**q** Horizon III. Flake cores: **a**–**j**; Bladelet cores: **k**–**q**. Bipolar: **F**, **H** Freehand knapping: **a**–**g**, **i**–**q**

Similar technological practices were observed in H-II (Fig. 8b, c, and e). While quartz remained the primary raw material type, a greater variability in reduction strategies is displayed compared to H-I. Cores were exploited on multiple surfaces, with removals initiated from plain and cortical platforms. The average number of removals for flake quartz cores is still low 2.5, and short last removals 22 mm (Table 5).

Cores in H-III accounted for 2.5% of the horizon assemblage, mostly made on quartz 50%, followed by chert 33.3% and quartzite 16.7% (Table 4). Quartz cores were for simple flake production using freehand knapping technique on a single flaking surface. The cores exhibited mostly a single removal of 66.6% of the quartz cores from unprepared cortical platforms. The cores were discarded with high cortical coverage (Table 4. Figure 8g and j).

Chert cores were primarily oriented toward micro-laminar production and were exclusively reduced using freehand knapping techniques. Reduction focused on a primary flaking surface, executed in a semi-rotational manner from a single plain platform (Fig. 8). Core exploitation followed either a unidirectional strategy, resulting in cortical backs remaining unexploited (55.6%), or an orthogonal strategy (60%). The cores were typically discarded once step fractures accumulated on the flaking surface (Fig. 8k–q).

### Modified Artifacts

**Fig. 9:**
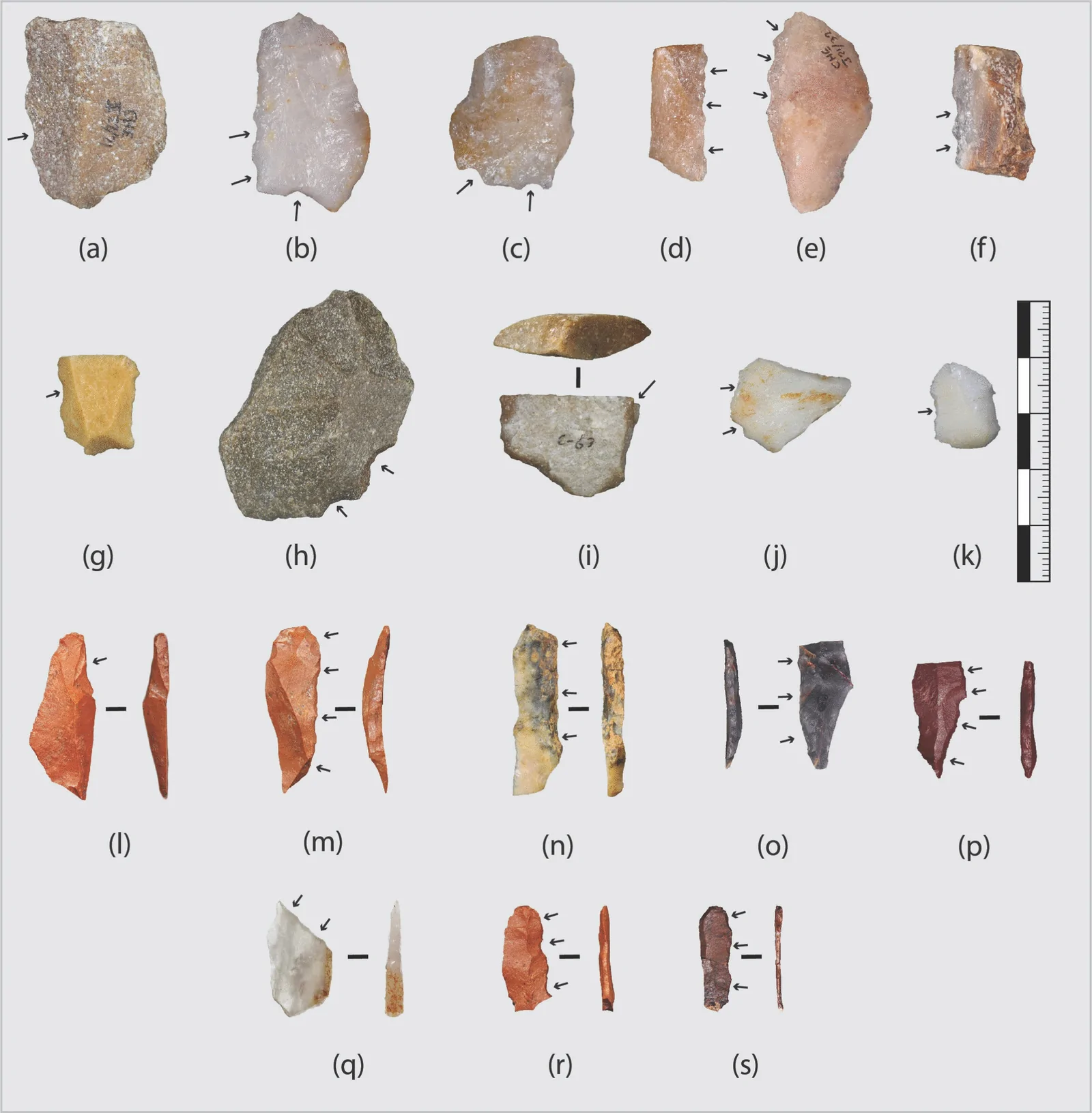
Selected tools from Chessungalane. **a**–**d** Horizon I; **e**–**f** Horizon II; **g**–**r** Horizon III. **l**–**r** Backed bladelets; **i** Burin; **a**–**h**, **j**–**k** Notch. *Note*: artifacts are oriented platform-up

Tools are present in low amounts, accounting for 1.3% of the total assemblage (Table 3; Fig. 9). In the lower horizons (H-I, H-II), tools were extremely rare, making up less than 1% of each horizon’s assemblage, limited to notches on quartz flakes. There is a notable increase in tool quantity and diversity in H-III, where tools accounted for 1.6% of the assemblage. Quartz flakes were mainly shaped into scrapers and truncations, alongside one fragmented backed piece. Quartzite flakes were either notched or modified with simple retouch. Chert bladelets were selected exclusively for backed tools ranging average thickness from 4.6 to 3 mm. The backed bladelets do not show a major modification in shape, rather straight retouch without geometric shapes.

### Shatter Fragments

The highest number of excavated artifacts in CHE were shatter and debris, making up 64.8% of the whole assemblage. The highest percentage of shatter was recorded in H-I with 78.4% and in H-III with the lowest yest high 57.4% predominantly on quartz (Table 3). High amounts were expected as a byproduct of quartz brattle fracturing.

### Refit Lithic Sequences

The highest percentages of refits were observed in H-III, with chert accounting for 77.6% and quartzite of 15.7%. In contrast, quartz exhibited low refit percentage (Table 6). A total of 22 refit sequences were reconstructed at CHE (Table 7). BR types were limited to four sets of fragmented flakes and bladelets across all raw materials, primarily from H-III, with the exception of two quartzite flake fragments from H-II. PSR type dominated the refitting, comprising 18 sequences, most of which were predominantly on chert. Only refit sets containing more than five artifacts are reported here, as they provide more substantial interpretive value; the remaining sequences are included in the supplementary materials.

**Table 6:** Percentage distribution of refitted artefacts by horizon and raw material, calculated as a proportion of the total number of refitted pieces

Horizon	Chert	Quartz	Quartzite	Total
I	-	2	1	3 (2.2%)
II	-	-	2	2 (1.5%)
III	104	7	18	129 (96.3%)
Total	**104 (77.6%)**	**9 (6.7%)**	**21 (15.7%)**	**134 (100%)**

**Table 7:** Spatial distribution of refitted artifact sets

Refit #	Area(s)	Horizon(s)	Raw material	Set(s)	Total Number	Avg_Horizontal	Avg_Vertical	Moran_I	P_Value
1	CON	III	Quartzite	PSR	8	61.4	4.5	− 0.06	0.3
2	CON	III	Quartz	PSR	3	56.6	14.3		
3	CON	III	Chert	PSR	8	92.4	8.3	− 0.01	0.11
4	CON	III	Chert	PSR	16	68.2	4.7	**0.42**	**0**
5	CON	III	Chert	PSR	19	37.6	4.2	**0.52**	**0**
6	CON	III	Chert	PSR	9	57.2	3.6	− 0.34	0.9
7	CON	III	Chert	PSR	7	73.5	5	0	0.1
8	CON	III	Chert	PSR	10	93.1	5.5	0.1	0.09
9	CON	III	Quartzite	PSR	7	83.6	5.9	− 0.13	0.38
10	CON	III	Chert	PSR	20	60	7	**0.37**	**0**
11	CON	III	Quartz	BR	2	32.2	13.8	-	-
12	CON	III	Chert	PSR	3	72.7	17.5	-	-
13	CON, TR	I, III	Quartzite	PSR	2	270.5	89.7	-	-
14	CON	III	Chert	PSR	2	76.1	11.6	-	-
15	TR	I	Quartz	PSR	2	280.5	24.3	-	-
16	TR	III	Quartzite	PSR	2	97	24.1	-	-
17	IJ	II	Quartzite	BR	2	37.5	0.5	-	-
18	CON	III	Quartz	PSR	2	47.9	5.5	-	-
19	CON	III	Chert	BR	2	20.9	0.6	-	-
20	CON	III	Chert	PSR	2	28.4	10.1	-	-
21	CON	III	Chert	BR	2	-	-	-	-
22	CON	III	Chert	PSR	2	22.4	12.9	-	-

### Laminar Core Refits

**Fig. 10:**
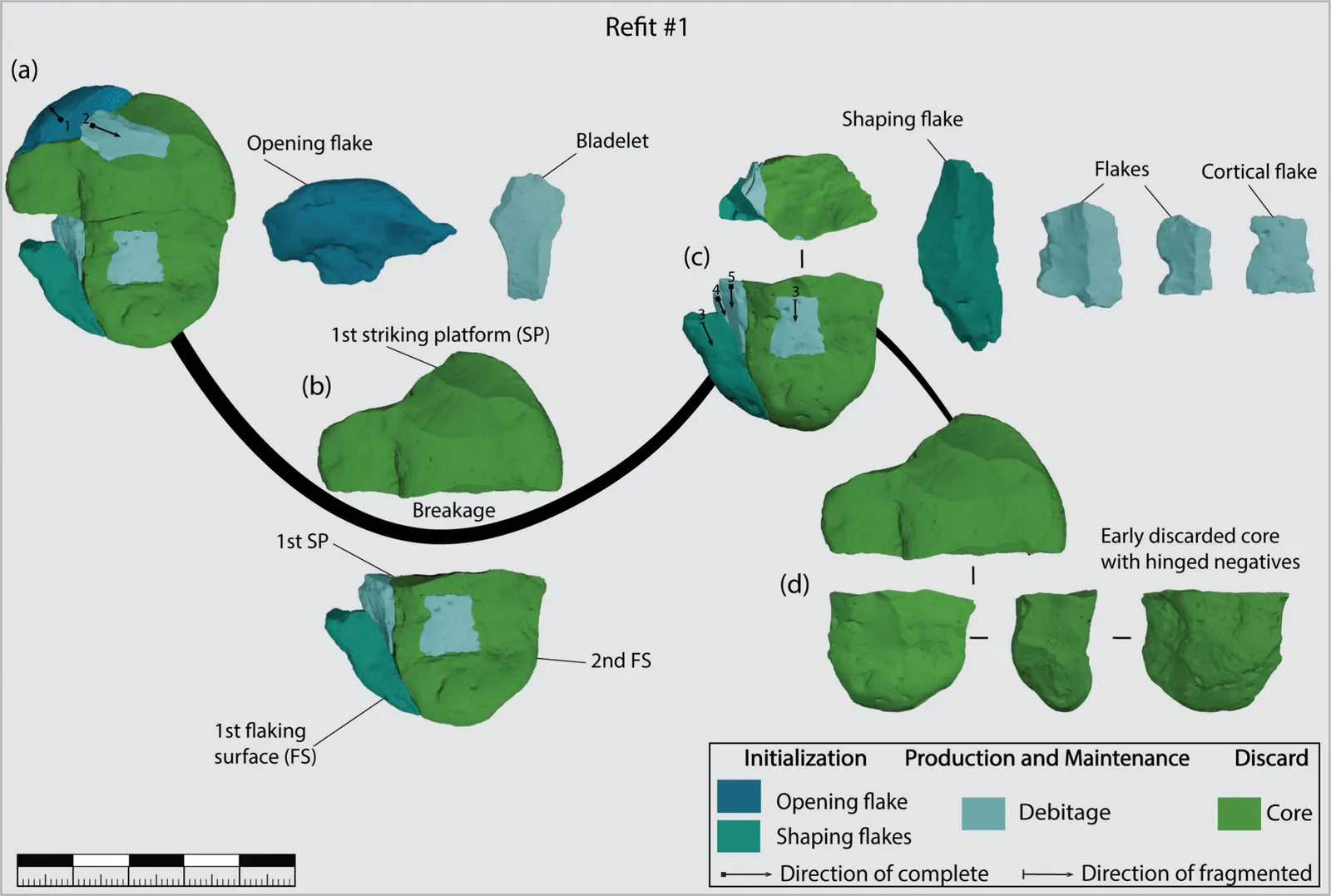
Refits diagram for the refit #1. 3D models were generated using Agisoft, illustrations on Blender (3D models are available in SI) (**a**–**d**)

**Refit #1** comprises two cores and eight quartzite artifacts recovered from H-III. The sequence shows unidirectional laminar production from single platform. The reduction sequence began with the removal of a cortical flake from one side of a pebble, creating a platform that enabled the detachment of relatively elongated flakes perpendicular to the core’s axis of symmetry (Fig. 10a). Subsequently a fracture, likely caused by geological flaws, split the core into two fragments (Fig. 10b).

Reduction continued on one of the fragments, using the naturally flat fracture surface as a striking platform, and taking advantage of the fragment’s natural convexity (Fig. 10c). This phase started with the removal of a cortical bladelet and a blade from the narrow natural surface, followed by an overlapping flake detached from the flat side. The last two removals ended in hinge fractures, resulting in the loss of the surface convexity (Fig. 10d). Although the core retained further potential reduction, it was discarded at this stage.

**Fig. 11:**
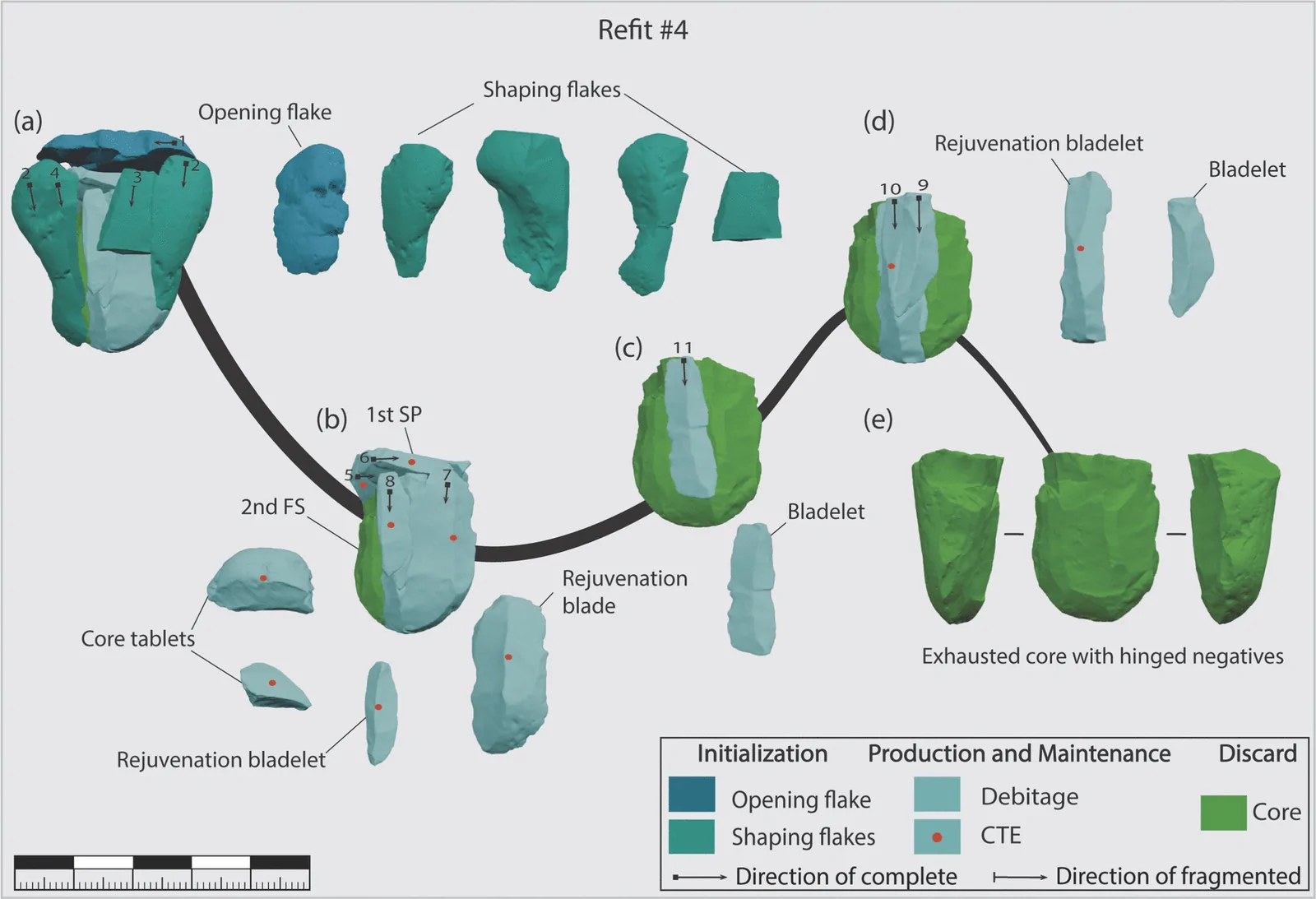
Refits diagram for refit 4. 3D models were generated using Agisoft, illustrations on Blender (3D models are available in SI) (**a**–**d**)

**Refit #4** includes a core and 15 chert pieces from H-III. The sequence represents a unidirectional laminar reduction from a single striking platform. The reduction began with the removal of an opening flake from the natural flat surface of the pebble, which terminated in a horizontal Siret fracture. From a plain platform, thick cortical blades were detached from opposing margins of the core, shaping a narrow, elongated flaking surface with a convex profile suitable for unidirectional bladelet production (Fig. 11a).

Only a few bladelets appear to have been removed after the shaping stage, but these are absent from the assemblage. To optimize the platform angle and facilitate further reduction, two core tablets were removed (Fig. 11b). This preparation enabled the detachment of a single bladelet from the rejuvenated platform (Fig. 11c). Subsequent removals, however, included an overpassed bladelet and a hinged bladelet, both terminating in hinge fractures (Fig. 11d). Attempts to rejuvenate the surface resulted in a series of hinge-terminated removals (Fig. 11e). The combination of the small core volume and repeated knapping errors rendered further reduction unviable, leading to the abandonment of the core.

**Fig. 12:**
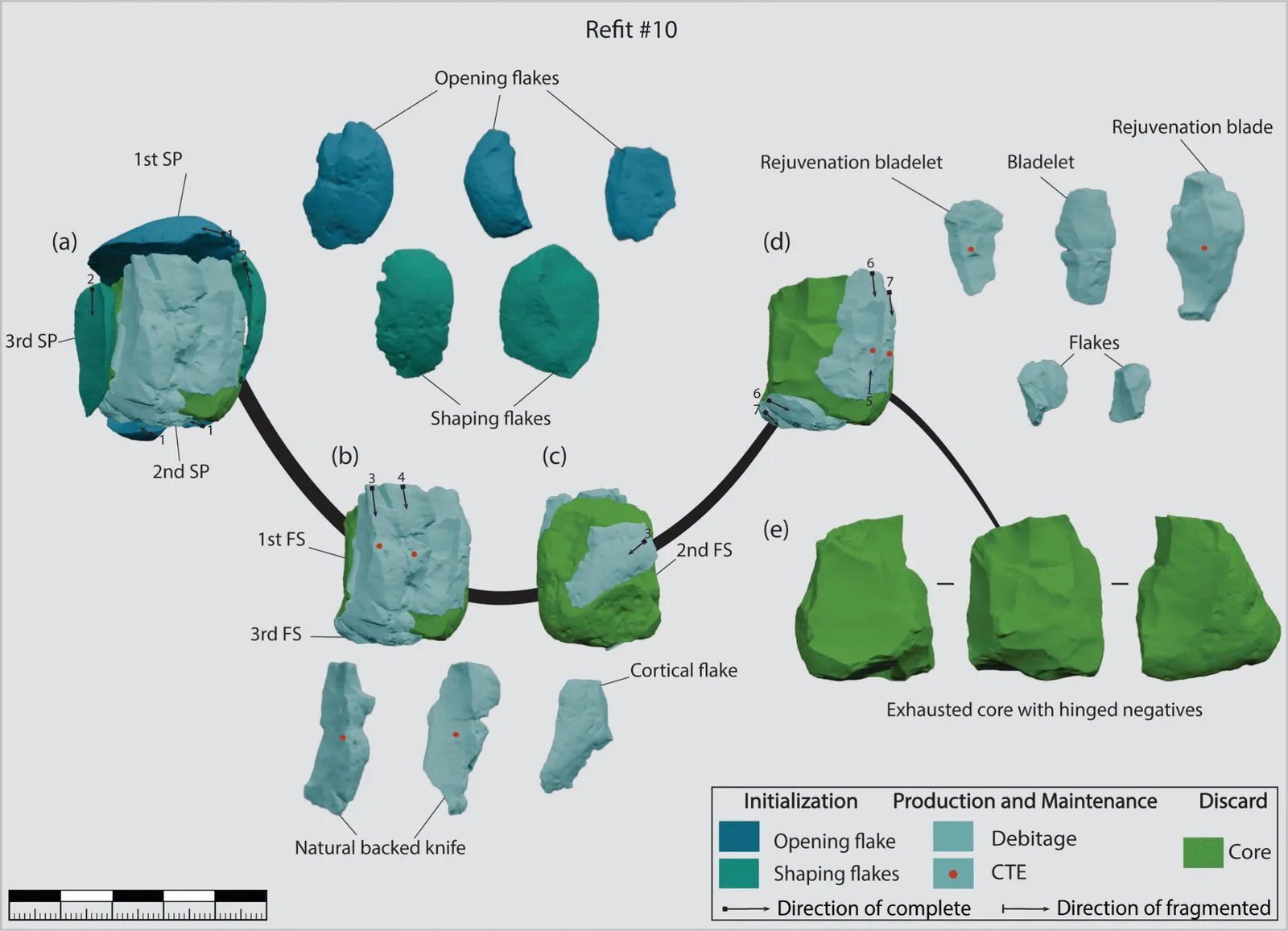
Refits diagram for refit #10. 3D models were generated using Agisoft, illustrations on Blender (3D models are available in SI) (**a**–**e**)

**Refit #10** consists of a core and 19 chert pieces from H-III, representing an orthogonal laminar reduction sequence from multiple striking platforms. Reduction began with the detachment of flakes from opposite axial ends of the pebble, creating two opposed platforms used to exploit a single flaking surface (Fig. 12a). To shape this surface, two elongated cortical and semi-cortical flakes were removed from the core margins via the first platform, which in turn created a third platform for further exploitation (Fig. 12a).

A few early decortication flakes appear to have been removed mainly from the primary platforms but are missing from the refit. This was followed by the removal of two naturally backed blades from the first platform (Fig. 12b). The core was then rotated to exploit the back surface, with a single cortical flake detached from the flat third platform, intersecting the earlier flaking surface orthogonally (Fig. 12c). A rejuvenation bladelet was struck to remove the hinge of a missing bladelet from the first platform, followed by a short bladelet with a hinged termination (Fig. 12d). Two short flakes were then extracted from the third platform on the same flaking surface.

The sequence concluded with another rejuvenation attempt, which again resulted in a hinge termination (Fig. 12d). Although the discarded core still retained unexploited volume, it was abandoned due to the reduced dimensions of the main platform and the loss of convexity along the longest axis, both of which would have hindered systematic laminar production (Fig. 12e).

### Flake Core Refits

**Fig. 13:**
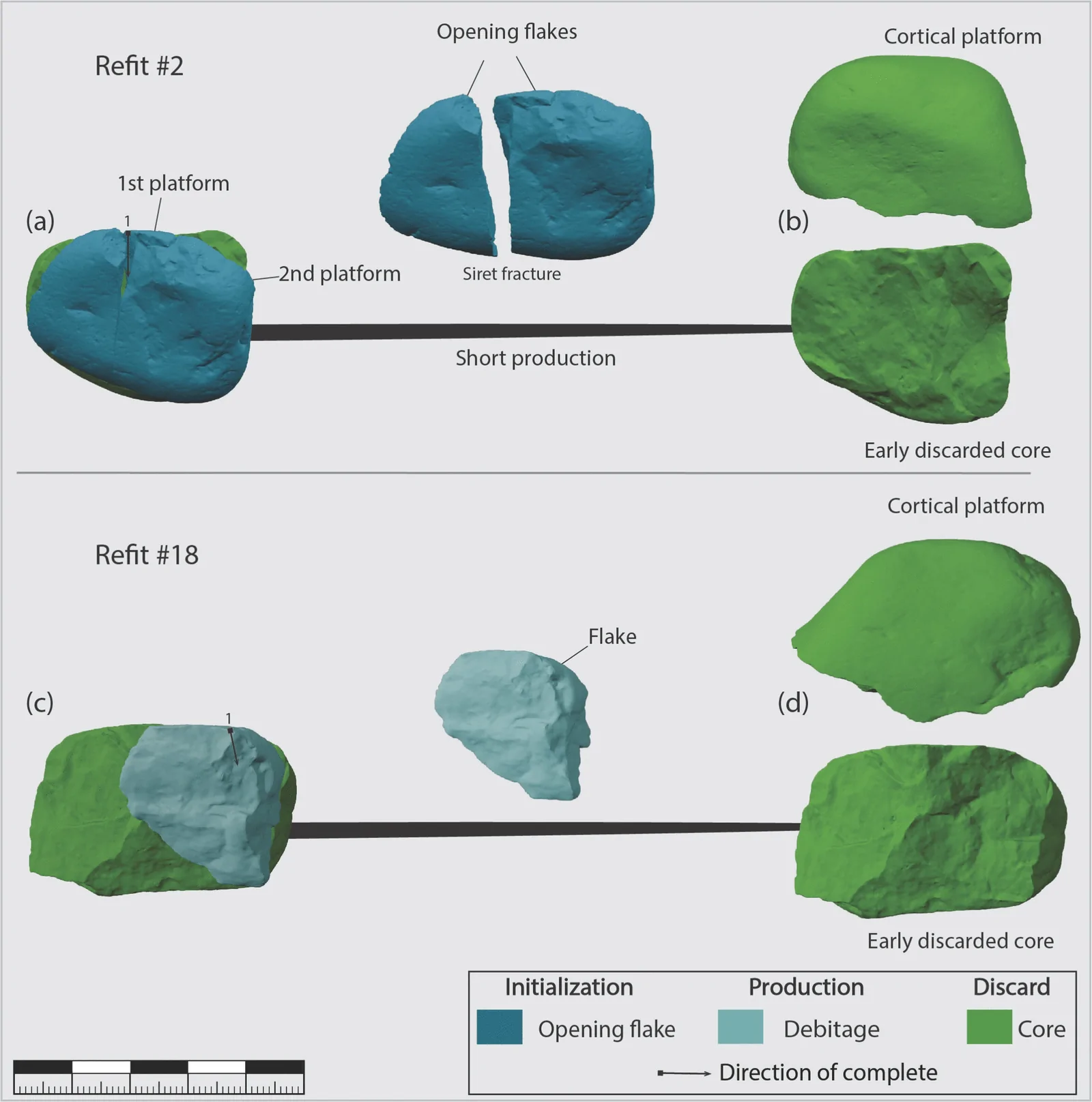
Refits diagram for refits 2 and #18. 3D models were generated using Agisoft, illustrations on Blender (**a**–**d**)

Only two core refits on quartz were identified, both representing flake production from unprepared platforms by freehand knapping. Refit #2 is a core, and two flakes began with the removal of a cortical flake that terminated in a longitudinal Siret fracture, followed by a short sequence from the second platform that is missing in the refit. Refit #18 preserves only the core and a single flake removed from a cortical platform. In both cases, the cores were discarded at an early stage despite retaining considerable unexploited volume (Fig. 13a-d).

Spatial analysis of 22 refits shows that horizontal displacements are generally greater than the vertical (SI, Table S4). Vertical movements are more limited, typically below 20 cm with the exception of Refit 12 (17.5 cm) and 13 (89.7 cm). Moran’s I analysis on refits with more than three artifacts show significant clustering for Refits 4, 5, and 10 (*p* = *0.00*), suggesting non-random spatial grouping. while refits 1, 3, 6, 7, 8, and 9 are randomly or non-significantly dispersed. Figure 12 shows the vertical distribution of the laminar refits (R-1, R-4, and R-10) in CON.

## Discussion

### Raw Material and Technological Organization at CHE

The lithic technology at CHE demonstrates that raw material property’s structure reduction strategies, tool design, and technological investment across the three occupation horizons. Rather than reflecting availability-driven choices alone, the differential treatment of quartz and chert reveals deliberate decisions about organising lithic production in relation to material quality and local resource abundance (Will, 2021).

### Quartz Technology: Low-cost Flake Strategy

**Fig. 14:**
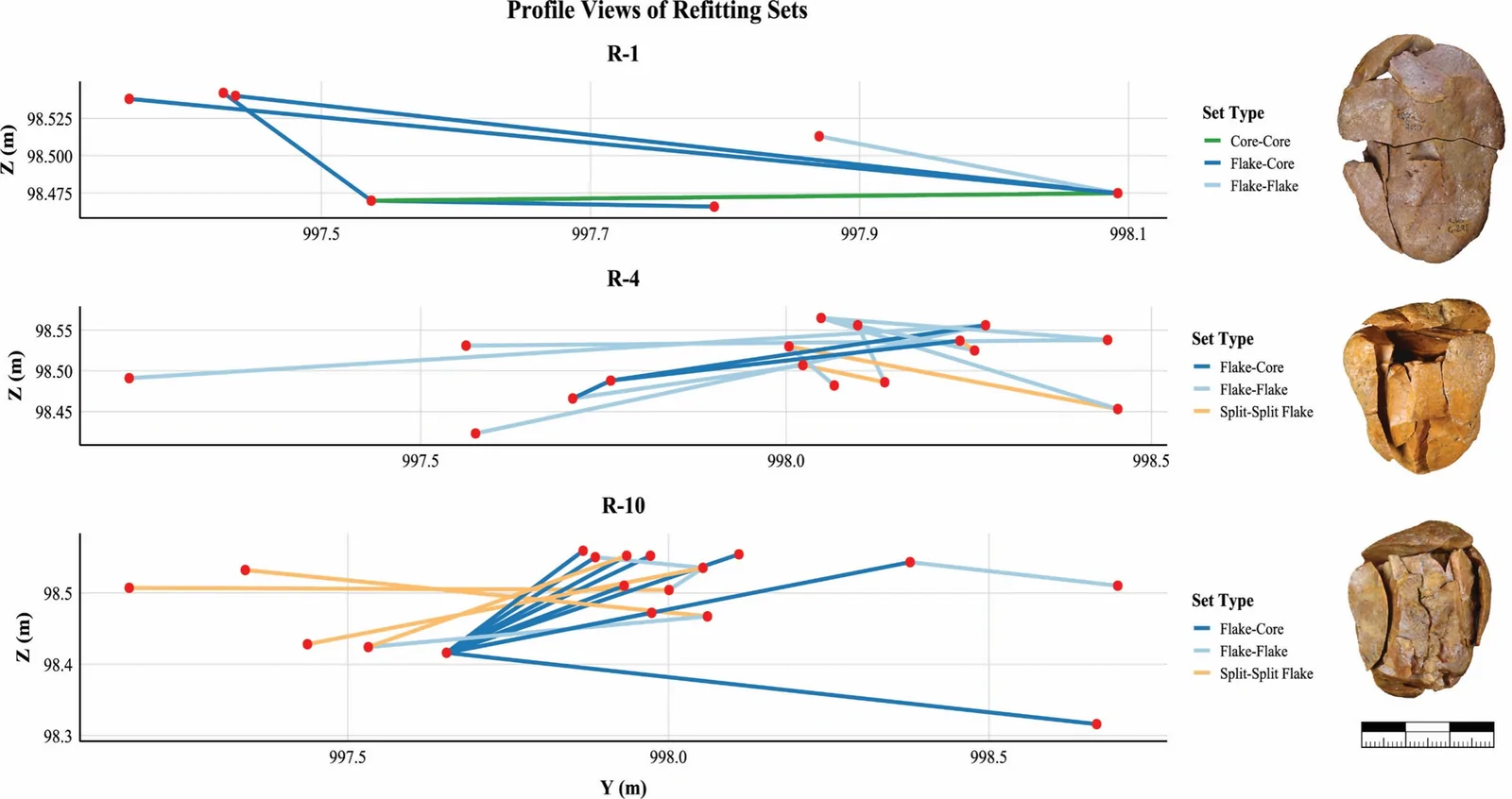
Chaîne opératoire reconstruction for chert bladelet production and quartz flake production at Chessungalane

The technological approach to quartz followed a low-investment strategy (Fig. 14). Knappers employed a simple *chaîne opératoire* comparable to the *tranche de saucisson* method documented in European Middle Palaeolithic contexts (Turq, 1989). This strategy produced flakes characterized by cortical platforms and steep lateral cross-sections, direct by-products of working small-radius pebbles without geometric management.

Core morphology was neither managed nor optimised, and short reduction sequences meant knapping errors were rarely encountered. Because only a few flakes were removed from each core, there was less need to manage core geometry or deal with the platform preparation challenges that arise during extended reduction. This technological simplicity reflects a rational economic decision: quartz was abundant, immediately available, and adequate for producing sharp-edged flakes for daily use. The simple nature of quartz exploitation is further evidenced by consistently low reduction intensity compared to other raw materials (Fig. 14).

**Fig. 15:**
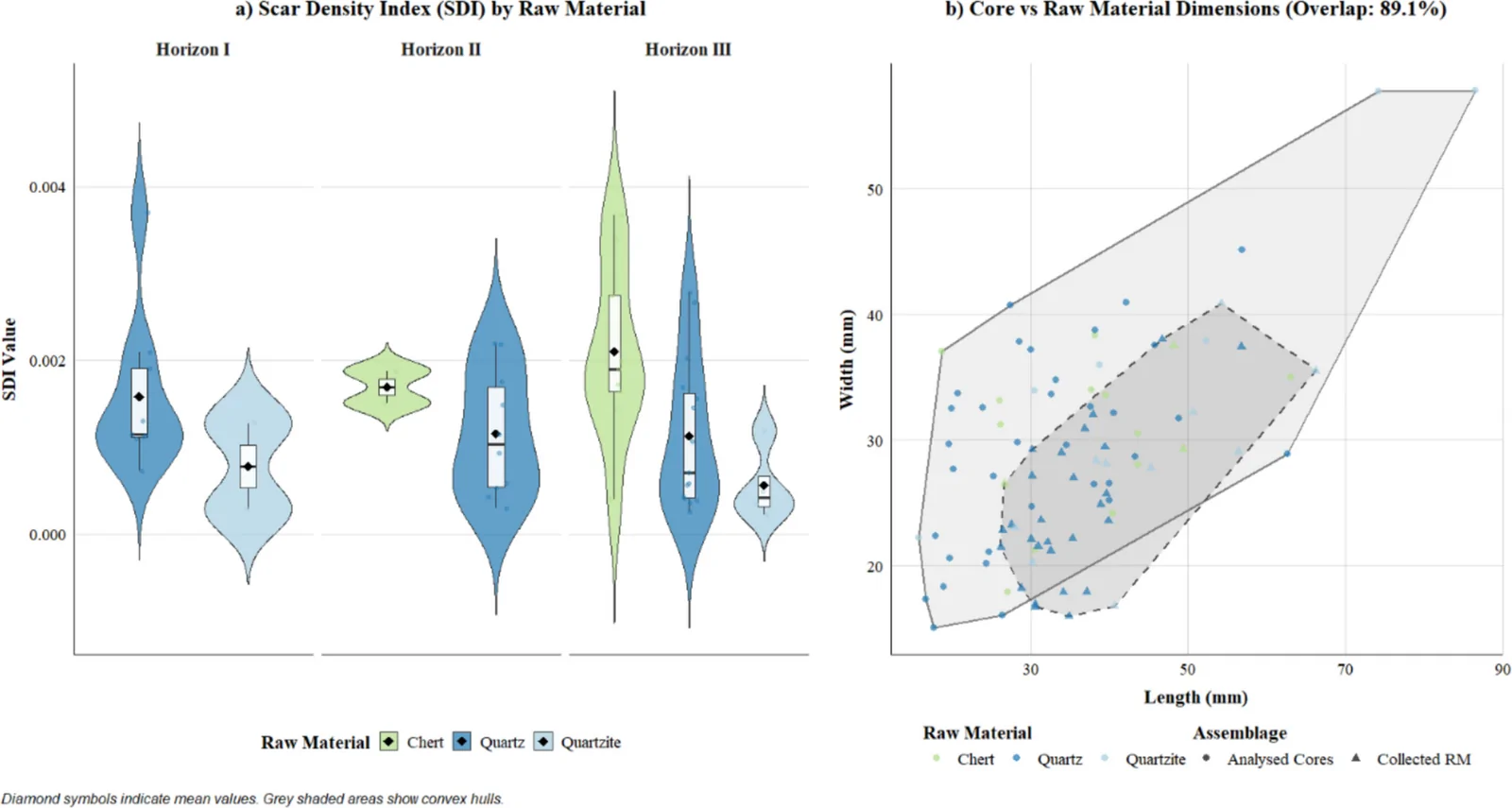
Reduction intensity and surveyed raw material at Chessungalane. **a** Scar Density Index (SDI) distributes by raw material and horizon showing differential reduction in intensities. **b** Comparison of discarded core dimensions versus collected raw material pebbles from surface survey

The knapping technique employed at CHE was predominantly freehand across all raw materials. Despite the known pattern of bipolar reduction on quartz as an adaptation to raw material constraints (Braun et al., 2009; Diez-Martín et al., 2011), or economic solution (Pargeter, 2016; Pargeter & de la Peña, 2017), this pattern was rarely adopted in CHE. The local abundance of quartz pebbles likely removed the economic incentive for bipolar reduction, as knappers could readily acquire new raw material rather than intensively exploit individual volume. This is further supported by the early discard of cores with high cortical coverage (Fig. 7) and low SDI (Fig. 15), indicating minimal investment in core reduction before abandonment. The frequent use of freehand knapping may therefore reflect both the absence of raw material pressure and inherited technological traditions. However, the high fragmentation rate (Table 3) in the quartz assemblage may have hindered the identification of bipolar characteristics, as these were securely identified only on complete artefacts.

The differential usage of raw materials extended into toolkit composition. During the earlier occupations (H-I and H-II), the toolkit remained simple and followed raw material availability: tools were made predominantly on quartz and quartzite, consisting of notches and scrapers.

### Chert Technology: High-cost Bladelet Strategy

Chert was used for micro-laminar production, likely because of its economic advantages (Eren et al., 2008). Despite being the least common raw material in local gravels, chert received the highest technological investment (Fig. 14). After initial cortical removal, chert cores were structured through platform preparation rather than opportunistic exploitation of pebble surfaces. Narrow flaking surfaces were established via elongated lateral removals (i.e., crested blades), creating the geometric conditions necessary for systematic micro-laminar production. When knapping errors occurred, knappers attempted rejuvenation through invasive removals or establishment of secondary platforms, actively extending core use-life. Higher SDI values on chert cores quantitatively confirms this intensive exploitation.

Percussion techniques for chert display greater organization than those used for quartz. While a strong relationship between percussor type and platform lipping remains debated (Driscoll & García-Rojas, 2014; Schindler & Koch, 2012), the possibility of soft hammer usage, indicated by lipped platforms combined with diffuse bulbs of percussion (Table 4), cannot be entirely refuted (Mourre et al., 2010). Knappers would have had to use a semi-soft hammerstone such as sandstone, or a soft hammer such as wood or bone, as no cervids are present in Africa (Moos et al., 2025).

A marked shift in toolkit composition appears in the final occupation horizon (H-III), where chert bladelets were selected exclusively for backed tools. This pattern suggests either an adaptive shift in technological strategy from earlier occupations (Binford, 1980) or the introduction of new groups with different technological knowledge. In addition, some naturally backed quartz flakes may have served as projectile weaponry, though without traceological evidence this remains conjecture.

**Fig. 16:**
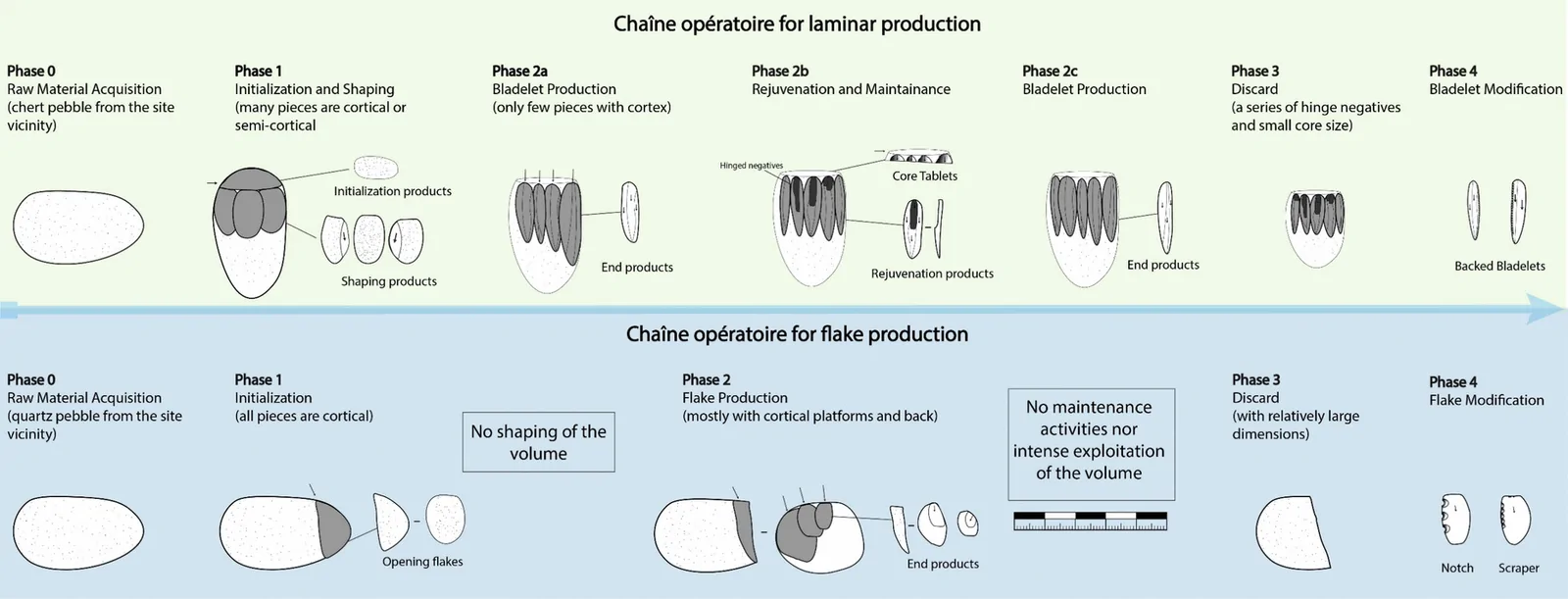
Vertical distribution of refits numbers 1, 4, and 10 in excavation area CON at Chessungalane

From an organizational perspective, both chert and quartz assemblages at CHE include opening flakes and ridge flakes, which appear broadly similar in terms of morphology (Figs. 11, 12, 13, 16). Yet their functional role within reduction strategies diverges. In chert, opening flakes were extracted to establish plain platforms, while ridge flakes actively shaped the flaking surface. In quartz, by contrast, opening flakes primarily represent initial production rather than platform preparation, and ridge flakes derive largely from the natural curvature of the pebble. This comparison highlights how raw material properties influenced reduction strategies, producing flakes with superficially similar typologies but fundamentally different functional significance (Figs. 14).

### Spatial Integrity

The refitting analysis provides evidence for site formation processes beyond its technological insights. The overall refit success rate of 17% among the total diagnostic artifacts is relatively high for an open-air site (Laughlin & Kelly, 2010). More importantly, spatial analysis reveals consistently less vertical than horizontal displacement within excavation area CON (Table 6). This pattern supports minimal post-depositional disturbance and high stratigraphic integrity.

The differential refit success across raw materials (chert 77.6%, quartzite 15.7%, quartz 6.7%) primarily reflects fracture properties and geological variation affecting recognizability rather than differential disturbance. The consistently greater horizontal than vertical displacement across all materials confirms that stratigraphic boundaries represent meaningful temporal divisions, supporting the interpretation of technological change from H-I/H-II to H-III as real occupational sequences rather than artefacts of poor preservation. For Refit 12, the vertical distance remains within H-III and does not cross horizons boundaries, suggesting localized post-depositional movement. Refit 13, however, represents an extreme case among the 134 refitted artifacts at CHE, with a vertical displacement of nearly 90 cm connecting H-I and H-III. Several factors may account for this displacement, including bioturbation through root activity or human recording error. It should be noted, however, that Refit 13 is an isolated case and does not reflect the overall pattern; further site formation studies may help clarify, this issue.

### Raw Material Economy: Local Procurement and Selective Exploitation

Comparison between collected modern raw materials and the cores from CHE reveals the local character of lithic procurement at the site. Present-day gravels are dominated by quartz, followed by quartzite, with minimal chert representation, and proportions closely mirroring the archaeological assemblage composition. Convex hull analysis demonstrates substantial geometric overlap (ratio 0.891) between archaeological core dimensions and locally sampled raw materials (Fig. 13B), confirming local procurement rather than transport from distant sources.

The technological variability at CHE was influenced by both raw material properties and availability. Quartz, readily available on site, required minimal transport and showed minimal reduction intensity before discard, following Henry’s (1989) distance-decay model. In contrast, chert was also procured locally but showed higher intensity of reduction and investment, aligning with the relationship between raw material quality and technological organization described by Andrefsky (1994) and Kuhn (1994).

This pattern demonstrates that technological investment responds to raw material quality and scarcity rather than simply to distance from source, with abundant local materials receiving less intensive treatment while scarce high-quality materials receive more complex strategies (Andrefsky, 1994; Nelson, 1991). Knappers at CHE made economic decisions tailored to the specific properties and local availability of different lithic materials rather than applying a single undifferentiated procurement-reduction system.

### CHE in the Context of the Regional LSA

The LSA archaeological record in Mozambique remains limited, with very few sites providing dated cultural sequences accompanied by detailed techno-typological analyses. In the north of the country, the Chicaza rock shelter in Niassa Province contains three stratified layers, two of which are affiliated with the LSA (~ 19–6 Ka), encompassing the transition from the Early LSA to the Robberg and the Oakhurst chronologically (Bicho et al., 2016). Preliminary results indicate a dominance of micro-flake production across all layers, predominantly on local quartz (Bicho et al., 2016). This technological pattern closely parallels H-I at CHE (~ 23.3–10.6 ka), which spans a comparable chronological range and is similarly characterized by small quartz flake production.

In the south of the country, the Txina-Txina site in the Limpopo Basin provides other multi-period LSA sequence from Mozambique with preliminary lithic data across four units (Bicho et al., 2018a, 2018b, 2023). Unit C (~ 27–14 ka), which chronologically overlaps with CHE’s H-I, reports bladelet production using both freehand and bipolar knapping on quartzite and quartz. Units D (~ 14–7.5 ka) and E (~ 7.5 ka to the Iron Age) continue to show both flake and bladelet production. This differs markedly from CHE, where bladelet production is entirely absent throughout H-I and H-II and only appears in H-III (~ 8.6–6.7 ka).

In highland Lesotho, Melikane Rock shelter preserves an occupation pulse dated to ~ 27–23 ka cal BP, directly overlapping with the basal levels of CHE’s H-I (~ 23.3 ka) (Pazan et al., 2023). Layer 5 (< 23.8 ka cal BP) is marked by microlithic flake production on cryptocrystalline silicates with unprepared platforms using unidirectional or multidirectional exploitation strategies. Bladelets are also common, bladelet cores account for 28% of all cores, bipolar reduction remains significant (~ 44%), and backed tools are present.

In eastern South Africa, Iron Pig Rock Shelter in Mpumalanga provides a well-analyzed LSA sequence spanning ~ 13.7 to ~ 8.9 ka (Bader et al., 2020). Layers 5 (~ 13.7–13.2 ka) and 6 (~ 10.9 ka) are dominated by local hornfels and quartz, with freehand knapping more prevalent. Unlike CHE, 92% of cores at Iron Pig were oriented toward bladelet production with prepared platforms and lipping indicative of freehand knapping.

In eastern Eswatini, Siphiso Shelter provides a well-dated LSA sequence spanning ~ 15,000 to 2,000 BP (Barham, 1989). The assemblage is flake-dominated throughout with minimal platform preparation and retouched tools accounting for less than 1%. During the Oakhurst phase (~ 9500–7600 BP), Siphiso unusually maintains low-frequency bladelet production, whereas CHE’s contemporaneous horizons show no laminar component. Both sites share the same timing for the appearance of backed tools, restricted to the Wilton transition. Bipolar reduction dominates at Siphiso versus predominantly freehand knapping at CHE, and flake size at Siphiso decreases through the Holocene with shifting raw materials, whereas at CHE all products remain microlithic throughout, constrained by the consistently small size of locally available quartz pebbles.

Taken together, these comparisons reveal that bladelet production was present across much of Southern Africa, and even in southern Mozambique at Txina-Txina, from the late Pleistocene onward, yet it is entirely absent from CHE until H-III (~ 8.6–6.7 ka). While Robberg technology was present at Melikane by ~ 24 ka and bladelet-oriented cores dominated at Iron Pig by ~ 13 ka, CHE populations maintained an exclusively simple flake-based tradition with a parallel introduction of bladelet during the last occupation phase. The Limpopo Basin may represent the approximate southern limit of groups practicing laminar traditions during the late Pleistocene, with this adaptation not reaching the central or northern areas of Mozambique. Whether CHE’s distinct trajectory reflects raw material constraints, site function, or an independent technological tradition remains an open question. These comparisons are preliminary, and understanding the local trajectory of the LSA within Mozambique must remain a priority before more meaningful regional syntheses can be attempted.

## Conclusion

This study presents the first detailed technological and refitting analysis of a stratified LSA site in Mozambique, documenting human occupation spanning ~ 23–7 ka. Given the sites’ techno-typological characteristics and the availability of local raw materials, CHE appears to represent an ephemeral workshop site. The availability of knappable raw material, combined with proximity to the Save River, made CHE a strategic location for knapping and tool-production recurring visits. These occupations in CHE were likely short-lived, as raw material availability was closely tied to the seasonality of the alluvial fan system, which periodically supplied fresh gravels suitable for knapping.

Understanding raw material properties and availability is critical for reconstructing past technological organization (Bamforth, 1986; Henry, 1989; Marks et al., 1991), as raw material acquisition is the first physical step following the mental organization of lithic production (Sellet, 1993). The locally available quartz pebbles at CHE constrained blank dimensions, resulting in microlithic assemblages. However, technological investment varied by raw material: quartz underwent simple, short reduction sequences with minimal core preparation, while chert in H-III received systematic bladelet production with higher investment by knappers. This demonstrates that raw material properties influenced both the scale of production and the degree of technological investment.

Our results reveal that technological organization in the LSA responds to multiple intersecting factors: environmental context shapes raw material availability, raw material properties constrain production scales, and social transmission maintains technological traditions across diverse material contexts. Combining OSL chronology with lithic analysis and refitting studies, this study establishes a foundation for future comparative research and highlights the need for continued investigation of southeastern Africa’s LSA record.

## Supplementary Information

Below is the link to the electronic supplementary material.

ESM 1 (DOCX 319 KB)

## Data Availability

The lithic dataset, refits dataset, OSL dating measurements, 3D models, used R code, and supplementary materials supporting this study are openly available at the Open Science Framework (OSF): https://osf.io/tyv2u/. Analyzed lithic materials are temporarily housed at ICArEHB, Universidade do Algarve, Portugal, for ongoing study under agreement with Mozambican authorities, with final curation in Mozambique.
