# Supplementary material for: Lithic Techno-economics and OSL Chronology of the Later Stone Age at Chessungalane, Save River Valley, Mozambique
Source: Afr Archaeol Rev. 2026 Jun 6;43(3):495–523. doi: 10.1007/s10437-026-09659-7 (PMC13558347; doi:10.1007/s10437-026-09659-7)
Supplement: Supplementary file 1 [file 10437_2026_9659_MOESM1_ESM.docx]

Lithic techno-economics and OSL chronology of the Later Stone Age at Chessungalane, Save River Valley, Mozambique.

**OSL Supplementary Information**

OSL sampling tubes were opened under subdued orange light (Sohbati et al., 2017) and outer material was used for dose rate determination using high resolution gamma spectrometry (Murray et al., 1987, 2018). The obtained radionuclide concentrations were converted into dry beta and gamma dose rate components using the conversion factors given by Creswell et al. (2018) (Table S1). The dry beta and gamma dose rates to 180-250 µm quartz grains were corrected for moisture assuming that the life-time average water content was 15% of the measured saturation values from inner tube material (as measured using the syringe method, see Murray et al., 2021). These values are slightly higher (2-4 %) than the in-situ water content measured on inner tube material; we adopt these higher values to allow for section drying out during excavation and potential drying out of the tube material during transport and storage. The cosmic contribution was added to the dose rate based on Prescott and Hutton (1994) and for quartz an internal alpha dose rate from U, Th of 0.010±0.005 Gy/ka was assumed (Vandenberghe et al., 2008). For the calculation of the K-rich feldspar dose rates (also for 180-250 µm grains) an internal beta dose rate of 0.90±0.04 Gy/ka was used; this assumes an internal K concentration of 12.5±0.5% for the feldspar grains. A higher internal alpha dose rate for feldspar grains of 0.10±0.05 Gy/ka was assumed. Total quartz and feldspar dose rates are summarized in Table S2.

The inner material (not potentially light-exposed) was processed under dark room conditions to extract 180-250 µm pure quartz and K-rich feldspar grains; procedures are described in Murray et al. (2021). Quartz grains were mounted as large (8 mm diameter) multigrain aliquots on stainless steel discs and feldspar as small (2mm diameter) multi-grain aliquots on stainless steel cups. Luminescence signals were measured on Risø TL/OSL readers calibrated for both discs and cups using Risø calibration quartz batch 207 (Autzen et al., 2022). The quartz OSL signal is dominated by a strong fast component. Equivalent doses (D_e_) for quartz were measured using SAR with a preheat of 260°C/10s and a cut-heat to 220°C. All calculations were made using early-background subtraction. The purity of the quartz OSL signal was confirmed by OSL IR depletion ratios being close to 1.00 (Duller, 2003). A dose recovery test was performed using 6 aliquots per sample yielding an average value of 0.979±0.010 (n=48) which indicates that our chosen SAR protocol is suitable to measure a dose prior to heat treatment in these samples. Feldspar was measured using the pIRIR_50,290_ protocol as described in Buylaert et al. (2012) and resulting IR_50_ and pIRIR_290_ D_e_ values are given in Table S2. Outliers were removed from the dose distributions using the interquartile range criterion (IQR; Tukey, 1977). Resulting IR_50_, pIRIR_290_ and quartz OSL ages are also summarized in Table S2 with uncertainties quoted at 1 standard error.

The differential bleaching rates of IR_50_, pIRIR_290_ and quartz OSL signals allow us to make a qualitative statement about how confident we can be that the quartz OSL signal is sufficiently bleached (Murray et al., 2012). Because the quartz OSL ages is consistent with the pIRIR_290_ age for sample OSL3 (252324) we can be confident that the quartz OSL signal was well-bleached at deposition; for the other samples we cannot be confident. Note: this does not mean that the quartz OSL was not sufficiently bleached at deposition, we just cannot be confident about this. At least from the stratigraphic consistency it appears that most quartz OSL ages (except OSL4) are reliable.

**Lithic Supplementary Information**

Table S3 Used attributes for the analysis.

|  | Attributes | Category | Description |
| --- | --- | --- | --- |
| 1 | Raw Material Type | Chert | Fine-grained siliceous rock |
|  |  | Quartz | Crystalline silica with variable knapping quality. |
|  |  | Quartzite | Metamorphosed sandstone, coarse and durable |
|  | Raw Material Transparency | Transparent | Light passes through clearly |
|  |  | Semi-Transparent | Light passes through partially |
|  |  | Opaque | No light transmission |
|  | Raw Material  Quality | Fine | Homogeneous, good knapping quality. |
|  |  | Medium | Minor impurities, workable |
|  |  | Impure | Visible flaws affecting knapping |
|  |  | Coarse | Large grains, poor knapping control |
| 2 | Completeness | Complete | Entire piece preserved. |
|  |  | Medial | Middle segment only. |
|  |  | Distal | Terminal end of the piece |
|  |  | Proximal | The top part of the piece with platform |
|  |  | Siret | Longitudinally broken piece |
| 3 | Knapping Technique | Freehand knapping | Direct percussion without support |
|  |  | Bipolar Knapping | Percussion on an anvil |
| 4 | Scars Direction | Uni-direction | Scars from one platform direction |
|  |  | Bi direction | Scars from two opposite platforms. |
|  |  | Lateral direction | Scars from the side |
|  |  | Cross direction | Intersecting scar directions |
|  |  | Orthogonal | Scars at right angles |
|  |  | Radial | Scars radiating from a central point. |
|  |  | Unk | Direction not identifiable |
| 5 | Remaining Cortex | 0% | No cortex present. |
|  |  | <50% | Partial cortex coverage. |
|  |  | >50% | Majority cortex coverage |
|  |  | 100% | Entire surface cortical |
| 6 | Profile | Flat | Straight longitudinal profile. |
|  |  | Curved | Strongly arched profile |
|  |  | Slightly Curved | Mild curvature. |
| 7 | Cross section | Cortical | Cortex present in section. |
|  |  | Flat | Even thickness |
|  |  | Lateral-steep | Steep edges on one side |
|  |  | Triangular | Three-sided section |
|  |  | Trapezoidal | Four-sided, uneven angles |
| 8 | Platform | Plain | Flat platform |
|  |  | Crushed | Damaged or shattered platform. |
|  |  | Cortical | Cortex-covered platform. |
|  |  | Facted | Prepared with multiple facets. |
|  |  | Lineal | Narrow, linear platform |
|  |  | Punctiform | Very small point platform |
|  |  | Missing | Platform not preserved |
| 9 | Lipping | Present | Lip formed below platform |
|  |  | Absent | No lip observed. |
| 10 | Radial Cracks | Present | Cracks radiating from impact. |
|  |  | Absent | No radial cracks |
| 11 | Point of impact | Present | Visible impact point. |
|  |  | Absent | Not visible. |
| 12 | Distal termination | Feather | Thin, smooth termination. |
|  |  | Hing | Rounded, abrupt termination |
|  |  | Overshot | Removal extends beyond core edge. |
|  |  | Irregular | Uneven termination |
|  |  | Modified | Retouched after removal. |
|  |  | Pointed | Sharp, pointed end |
|  |  | Step | Abrupt stepped termination |
| 13 | Cores platform dimensions | Platform Width | Maximum platform breadth. |
|  |  | Platform Depth | Platform thickness |
| 14 | Number of removals | Numeric | Count of removals. |
| 15 | Number of Mistakes | Numeric | Failed or flawed removals |
| 16 | Last removal length | Numeric | Max length of the last removal negative larger than 1cm |
| 17 | Bulb of percussion | Diffused | Weak or spread bulb |
|  |  | Pronounce | Strong, clear bulb |
|  |  | Fragmented | Broken bulb area |
|  |  | N/A | Not applicable. |
| 18 | Dimensions | Max length |  |
|  |  | Max width |  |
|  |  | Max thickness |  |
|  |  | Weight |  |
| 19 | Core form | Cylindrical | Elongated, rounded shape |
|  |  | Flat | Broad and thin |
|  |  | Polyhedral | Multiple flaking surfaces. |
|  |  | Spheroidal | Rounded, globular form |
| 20 | Core Type | Blade Core | Elongated and wide flaking scars on the core surface |
|  |  | Bladelet Core | Elongated and narrow flaking scars on the core surface |
|  |  | Flake Core | Oval flaking scars on the core surface |

Table S4 Spatial distribution of refitted artifact.

| **Refit #** | **Min_Horizontal** | **Max_Horizontal** | **Avg_Horizontal** | **Min_Vertical** | **Max_Vertical** | **Avg_Vertical** | **Moran_I** | **Expectation** | **Variance** | **Std_Dev** | **P_Value** |
| --- | --- | --- | --- | --- | --- | --- | --- | --- | --- | --- | --- |
| 1 | 16.6 | 155.2 | 61.4 | 0.2 | 9.8 | 4.5 | -0.06 | -0.14 | 0.03 | 0.52 | 0.3 |
| 2 | 39.1 | 82.4 | 56.6 | 4.6 | 21.4 | 14.3 | NA | NA | NA | NA | NA |
| 3 | 3.9 | 206.1 | 92.4 | 0.3 | 26.9 | 8.3 | -0.01 | -0.17 | 0.02 | 1.24 | 0.11 |
| 4 | 2.9 | 157.9 | 68.2 | 0 | 14.2 | 4.7 | 0.42 | -0.07 | 0.03 | 2.96 | 0 |
| 5 | 0.9 | 171.3 | 37.6 | 0.1 | 12 | 4.2 | 0.52 | -0.06 | 0.02 | 3.79 | 0 |
| 6 | 1.3 | 133.8 | 57.2 | 0.1 | 7.6 | 3.6 | -0.34 | -0.13 | 0.03 | -1.28 | 0.9 |
| 7 | 9.6 | 153.2 | 73.5 | 0.2 | 15.8 | 5 | 0 | -0.17 | 0.02 | 1.3 | 0.1 |
| 8 | 3.6 | 262.7 | 93.1 | 0 | 14.9 | 5.5 | 0.1 | -0.1 | 0.02 | 1.33 | 0.09 |
| 9 | 20.4 | 214.8 | 83.6 | 0.5 | 13 | 5.9 | -0.13 | -0.17 | 0.01 | 0.31 | 0.38 |
| 10 | 2.9 | 173.7 | 60 | 0 | 24.3 | 7 | 0.37 | -0.06 | 0.02 | 3.22 | 0 |
| 11 | 32.2 | 32.2 | 32.2 | 13.8 | 13.8 | 13.8 | NA | NA | NA | NA | NA |
| 12 | 72.7 | 72.7 | 72.7 | 17.5 | 17.5 | 17.5 | NA | NA | NA | NA | NA |
| 13 | 270.5 | 270.5 | 270.5 | 89.7 | 89.7 | 89.7 | NA | NA | NA | NA | NA |
| 14 | 76.1 | 76.1 | 76.1 | 11.6 | 11.6 | 11.6 | NA | NA | NA | NA | NA |
| 15 | 280.5 | 280.5 | 280.5 | 24.3 | 24.3 | 24.3 | NA | NA | NA | NA | NA |
| 16 | 97 | 97 | 97 | 24.1 | 24.1 | 24.1 | NA | NA | NA | NA | NA |
| 17 | 37.5 | 37.5 | 37.5 | 0.5 | 0.5 | 0.5 | NA | NA | NA | NA | NA |
| 18 | 47.9 | 47.9 | 47.9 | 5.5 | 5.5 | 5.5 | NA | NA | NA | NA | NA |
| 19 | 20.9 | 20.9 | 20.9 | 0.6 | 0.6 | 0.6 | NA | NA | NA | NA | NA |
| 20 | 28.4 | 28.4 | 28.4 | 10.1 | 10.1 | 10.1 | NA | NA | NA | NA | NA |
| 22 | 22.4 | 22.4 | 22.4 | 12.9 | 12.9 | 12.9 | NA | NA | NA | NA | NA |

Figure S1 UpSet plots showing the co-occurrence of bipolar and freehand knapping diagnostic attributes among complete quartz and chert artifacts from Chessungalane.

**SI References**

Autzen, M., Andersen, C.E., Bailey, M., Murray, A.S., 2022. Calibration quartz: An update on dose calculations for luminescence dating. Radiation Measurements 157, 106828. https://doi.org/10.1016/j.radmeas.2022.106828

Buylaert, J.-P., Jain, M., Murray, A. S., Thomsen, K. J., Thiel, C., Sohbati, R., 2012. A robust feldspar luminescence dating method for Middle and Late Pleistocene sediments. Boreas 41, 435–451.

Cresswell, A.J., Carter, J., Sanderson, D.C.W., 2018. Dose rate conversion parameters: Assessment of nuclear data. Radiation Measurements 120, 195–201. <https://doi.org/10.1016/j.radmeas.2018.02.007>

Duller, G.A.T., 2003. Distinguishing quartz and feldspar in single grain luminescence measurements. Radiation Measurements 37, 161 - 165. DOI: 10.1016/S1350-4487(02)00170-1

Murray, A.S., Marten, R., Johnston, A. & Martin, P. Analysis for naturally occurring radionuclides at environmental concentrations by gamma spectrometry. Journal of Radioanalytical and Radiochemistry. 115, 263-288 (1987).

Murray, A. S., Thomsen, K. J., Masuda, N., Buylaert, J. P., Jain, M. 2012. Identifying well-bleached quartz using the different bleaching rates of quartz and feldspar luminescence signals. Radiation Measurements 47, 688–695.

Murray, A. S., Helsted, L. M., Autzen, M., Jain, M., Buylaert, J. P., 2018. Measurement of natural radioactivity: calibration and performance of a high-resolution gamma spectrometry facility. Radiation Measurements 15, 215–220.

Murray, A., Arnold, L.J., Buylaert, J.-P., Guérin, G., Qin, J., Singhvi, A.K., Smedley, R., Thomsen, K.J., 2021. Optically stimulated luminescence dating using quartz. Nat. Rev. Methods Prim. 1, 72. <https://doi.org/10.1038/s43586-021-00068-5>.

Prescott, J.R., Hutton, J.T., 1994. Cosmic ray contributions to dose rates for luminescence and ESR dating: Large depths and long-term time variations. Radiation Measurements 23, 497–500.

Tukey, J.W, 1977. Exploratory Data Analysis. Reading, Mass.: Addison Wesley

Vandenberghe, D., De Corte, F., Buylaert, J.-P., Kučera, J., Van den haute, P., 2008. On the internal radioactivity in quartz, Radiation Measurements 43, 771-775. https://doi.org/10.1016/j.radmeas.2008.01.016.
